# EIF3H Modulates Glycolysis Through LDHA Stabilization in Triple-Negative Breast Cancer

**DOI:** 10.3390/cancers18172735

**Published:** 2026-08-23

**Authors:** Xuyu Cheng, Xinghai Liu, Ziyu Feng, Xiaoan Liu

**Affiliations:** Department of Breast Surgery, The First Affiliated Hospital with Nanjing Medical University, Nanjing 210029, China; c13645186039@163.com (X.C.); xinghai.med@foxmail.com (X.L.); 15733189127@163.com (Z.F.)

**Keywords:** triple-negative breast cancer, EIF3H, glycolysis, LDHA, protein ubiquitination

## Abstract

Background: Triple-negative breast cancer (TNBC) lacks effective targeted therapies, and its dependence on glycolysis represents a potential metabolic vulnerability. EIF3H, the largest subunit of the eukaryotic translation initiation factor 3 complex and a putative deubiquitinase, has been implicated in tumor progression, but its role in TNBC metabolism remains unclear. Methods: EIF3H expression and prognostic value were evaluated in public datasets, clinical TNBC specimens, and cell lines. Functional roles were examined using proliferation, colony formation, migration, and xenograft assays. Mechanisms were investigated by mass spectrometry, co-immunoprecipitation, ubiquitination and glycolytic rate assays, macrophage co-culture, and single-cell transcriptomic analysis. Results: EIF3H was significantly upregulated in TNBC and associated with poor survival. Transcriptionally activated by TRPS1, EIF3H bound to lactate dehydrogenase A (LDHA), reduced its ubiquitination, and prevented its proteasomal degradation. LDHA stabilization enhanced glycolysis and lactate production, thereby promoting TNBC cell proliferation and migration in vitro and tumor growth in vivo; these effects were abolished by LDHA knockdown and restored by LDHA re-expression. In addition, tumor-derived lactate induced M2 macrophage polarization via GPR65, which in turn reinforced malignant progression. Conclusions: Our findings define a TRPS1–EIF3H–LDHA axis that drives glycolysis-dependent TNBC progression and reveal lactate–GPR65 signaling as a mediator of tumor–macrophage crosstalk, supporting EIF3H as a potential prognostic biomarker and therapeutic target in TNBC.

## Simple Summary

Triple-negative breast cancer is the most aggressive subtype of breast cancer, with limited treatment options and poor survival, largely because it lacks the targets used by hormone- or HER2-directed therapies. These tumor cells depend heavily on glycolysis, an energy-generating pathway that produces large amounts of lactate, to fuel their growth and spread. Here, we show that a protein called EIF3H is abnormally elevated in triple-negative breast cancer and is linked to worse patient outcomes. EIF3H protects a key glycolytic enzyme, lactate dehydrogenase A, from degradation, which drives up lactate production. This excess lactate acts on nearby immune cells called macrophages through the receptor GPR65, pushing them toward a state that favors tumor growth. Blocking EIF3H markedly slowed tumor growth in both cell and animal models. These findings point to a previously unrecognized metabolic mechanism behind this disease and position EIF3H as a promising target for precision therapy.

## 1. Introduction

Triple-negative breast cancer (TNBC) is widely regarded as the most aggressive breast cancer subtype, as tumor cells express neither estrogen receptor (ER) nor progesterone receptor (PR) nor human epidermal growth factor receptor 2 (HER2) [1,2]. Although TNBC accounts for a relatively small proportion of all breast cancer cases, it is associated with exceptionally high mortality, limited therapeutic options, and generally poor prognosis [3,4]. While advances in targeted therapies against estrogen and HER2 have markedly improved outcomes in other breast cancer subtypes, chemotherapy remains the primary treatment for TNBC. Consequently, the identification of effective precision-targeted therapies with minimal adverse effects is of substantial clinical importance [5,6].

Metabolic reprogramming is a well-established hallmark of cancer, characterized especially by the preferential use of aerobic glycolysis, known as the Warburg effect, which facilitates tumor proliferation, migration, and invasion [7,8,9]. In advanced stages, such metabolic alterations may contribute to cachexia through increased glucose consumption [10,11,12]. Pyruvate kinase M2 (PKM2) and lactate dehydrogenase (LDH), two central regulators of the Warburg effect, are commonly overexpressed in TNBC [13,14,15,16]. Targeting these metabolic enzymes may suppress glycolysis and thereby inhibit cancer progression. TNBC, like other breast cancer subtypes, exhibits elevated glucose uptake and lactate secretion; however, the specific contributions and implications of the Warburg effect in TNBC remain incompletely understood [17,18,19,20].

Ubiquitination and deubiquitination are critical post-translational modifications that play essential roles in cancer metabolism and have emerged as potential therapeutic targets [21,22]. Deubiquitinating enzymes (DUBs) have increasingly been recognized as key contributors to tumor initiation and progression. For example, USP22 enhances hypoxia-induced stemness in hepatocellular carcinoma through an HIF1α/USP22 feedback loop following TP53 inactivation, whereas USP25 regulates HIF-1-driven metabolic reprogramming in pancreatic cancer [23,24]. Accumulating evidence has demonstrated the anti-cancer potential of DUB inhibitors; the USP48 inhibitor DUB-IN-2 reduces HMGA2 levels to suppress colorectal cancer invasion and metastasis, while the UCHL1 inhibitor LDN-57444 attenuates neuroendocrine cancer progression [25,26].

Among the DUBs implicated in cancer, EIF3H (also known as eIF3a or p40) is an atypical member: it is the largest subunit of the eukaryotic translation initiation factor 3 (eIF3) complex and plays a central role in translation initiation [27,28]. Beyond this canonical function, EIF3H harbors a non-canonical MPN/JAMM domain that can confer deubiquitinase activity toward specific substrates [29]. EIF3H overexpression is common in human cancers and has been linked to tumor progression in hepatocellular carcinoma [29] and colorectal cancer [30], esophageal squamous cell carcinoma [31], melanoma [32], osteosarcoma [33], and thyroid cancer [34]. In breast cancer, amplification of 8q23.3, where EIF3H resides, together with EIF3H overexpression, has been reported in approximately 20% of untreated primary tumors [35]; more recently, EIF3H was shown to drive breast tumor invasion and metastasis by deubiquitinating and stabilizing YAP, and high EIF3H expression predicted poor recurrence-free survival in patients with basal-like breast cancer [36]. Despite these advances, whether EIF3H participates in the metabolic reprogramming of breast cancer, particularly the glycolytic dependence of TNBC, has not been explored.

In this study, we investigated EIF3H expression and its functional role in TNBC and identified EIF3H as an oncogene that is significantly associated with patient survival. Our results indicate that EIF3H promotes TNBC growth by stabilizing lactate dehydrogenase A (LDHA) and thereby enhancing LDHA-mediated glycolysis, which is essential for tumor progression and metastasis. Collectively, these findings provide mechanistic insights and support the development of therapeutic strategies targeting EIF3H to enable personalized treatment for TNBC.

## 2. Materials and Methods

### 2.1. Clinical Specimens and Immunohistochemistry

This study was conducted in compliance with the Declaration of Helsinki and approved by the Ethics Committee of the First Affiliated Hospital with Nanjing Medical University (approval no. 2025-SR-1092, approved on 26 November 2025). Human TNBC tissues and non-tumor controls were collected from the Breast Center of the First Affiliated Hospital with Nanjing Medical University, between August 2022 and February 2024. All patients signed informed consent before tissue specimen collection. Normal tissues were harvested from sites located more than 2 cm from the primary tumor. Histopathological diagnosis was confirmed by at least two independent pathologists according to the World Health Organization (WHO) classification criteria. Detailed patient characteristics are provided in Supplementary Table S1.

Paraffin-embedded tissue was sectioned at 3 μm and mounted onto slides using a microtome (Leica, Wetzlar, Germany) for immunohistochemical staining. Slides underwent deparaffinization using xylene and a graded series of alcohol. Following antigen retrieval in 10 mM citrate buffer (pH 6.0) at 95 °C for 20 min, sections were incubated overnight at 4 °C with primary antibody and then for 1 h with secondary antibody, and images were captured on a Thunder Imaging System (Leica). Two pathologists blinded to clinical information independently scored staining intensity (0, negative; 1, weak; 2, moderate; 3, strong) and the percentage of positive cells (0–100%); the H-score, ranging from 0 to 300, was obtained by multiplying these two values.

### 2.2. Cell Culture and Transfection

The normal breast epithelial line MCF-10A (CRL-10317) and breast cancer lines MDA-MB-231 (HTB-26), MCF-7 (HTB-22), and BT-549 (HTB-122) were purchased from ATCC (Manassas, VA, USA) and maintained in DMEM containing 10% fetal bovine serum (WISENT, Nanjing, China) and 1% penicillin/streptomycin (Gibco, Waltham, MA, USA) at 37 °C with 5% CO2. These lines represent three subtypes: basal-like (MDA-MB-231, BT-549), luminal (MCF-7), and normal mammary epithelium (MCF-10A). THP-1 monocytes (ATCC, TIB-202) were cultured in RPMI-1640 with 10% FBS and 1% penicillin/streptomycin and differentiated into M0 macrophages by 24 h treatment with 10 ng/mL phorbol 12-myristate 13-acetate (PMA).

Ubiquitination assays were performed by transiently co-transfecting cells with HA-tagged ubiquitin plasmids via Lipofectamine 3000 (Invitrogen, Carlsbad, CA, USA) following the manufacturer’s protocol, with cells harvested 48–72 h post-transfection.

Lentiviruses encoding EIF3H (OE-EIF3H) and short hairpin RNAs targeting TRPS1 (shTRPS1-1, shTRPS1-2, and shTRPS1-3), EIF3H (shEIF3H), LDHA (shLDHA), and GPR65 (shGPR65), as well as the corresponding negative controls (OE-NC and shNC), were constructed and packaged by GenePharma (Shanghai, China). Cells were infected according to the manufacturer’s instructions, and stably transduced cells were selected with puromycin. Overexpression and knockdown efficiencies were verified by Western blotting and qRT-PCR.

### 2.3. Bioinformatics Analysis

EIF3H mRNA expression across breast cancer molecular subtypes, and in TNBC relative to normal tissue, was examined in the TCGA-BRCA cohort together with the GSE76250, GSE65194, and GSE161529 datasets from the Gene Expression Omnibus (GEO). Overall survival (OS) and distant metastasis-free survival (DMFS) were assessed in the Kaplan–Meier plotter database and the TCGA-TNBC cohort, stratifying patients by median or optimal cutoff expression. Copy number alterations of EIF3H were retrieved from seven breast cancer datasets in cBioPortal. To identify transcription factors correlated with EIF3H (Pearson r > 0.3), we screened the TCGA-BRCA dataset through UALCAN and the METABRIC dataset through cBioPortal, and cross-referenced the overlapping candidates. The TRPS1 binding motif and its putative sites within the EIF3H promoter were predicted with JASPAR. Single-cell RNA-sequencing data from GSE161529 were processed and visualized in Seurat; cell-cell communication between tumor cells and macrophages was inferred with CellChat, and UMAP was used to visualize major cell populations and lactate receptor expression.

### 2.4. RNA Extraction and Quantitative Real-Time PCR (qRT-PCR)

Total RNA was extracted from cultured cells with an RNA extraction kit (Vazyme, Nanjing, China; cat. no. ROA3301-01) per the manufacturer’s instructions and reverse-transcribed into cDNA using a reverse transcription kit (Toyobo, Osaka, Japan; cat. no. FSQ-101). Quantitative PCR was performed in triplicate with SYBR Green master mix on an Applied Biosystems 7900 Fast Real-Time PCR System (Thermo Fisher Scientific, Waltham, MA, USA), using PPIA as the internal control; relative expression was calculated by the 2^−ΔΔCt^ method.

### 2.5. Fluorescence Microscopy

Cells were fixed with 4% paraformaldehyde (15 min), permeabilized with 0.5% Triton X-100 (30 min), and blocked with 5% BSA (30 min). Primary antibodies, diluted in Universal Antibody Dilution Buffer (cat. no. RM02955, ABclonal, Wuhan, China), were applied overnight at 4 °C, followed by secondary antibody incubation for 2 h at room temperature. Nuclei were counterstained with DAPI, and colocalization was imaged on a Leica TCS SP8 confocal microscope.

### 2.6. Dual-Luciferase Reporter Assay

To construct the reporter, the segment of the EIF3H promoter encompassing the putative TRPS1-binding motif was subcloned into pGL3-Basic (Promega, Madison, WI, USA). This construct was then co-delivered into MDA-MB-231 cells together with pRL-TK (internal reference) and either OE-TRPS1 or its corresponding empty-vector control, using Lipofectamine 3000 (Invitrogen, Carlsbad, XA, USA). Forty-eight hours post-transfection, firefly and Renilla luciferase signals were quantified with the Dual-Luciferase Reporter Assay System (Promega, USA), as previously described [37], with the former normalized against the latter.

### 2.7. Chromatin Immunoprecipitation (ChIP) Assay

ChIP assays were performed using a commercial ChIP assay kit (Beyotime, Shanghai, China). MDA-MB-231 cells were cross-linked with 1% formaldehyde for 10 min at room temperature, and the reaction was quenched with glycine. Chromatin was sonicated into fragments of 200–500 bp. Two percent of the total sheared chromatin was saved as the input control. The remaining chromatin was immunoprecipitated overnight at 4 °C with an anti-TRPS1 rabbit polyclonal antibody (polyclonal, Cat# 21938-1-AP, 1:100, Proteintech, Wuhan, China) or normal IgG. The immunocomplexes were captured with Protein A/G-agarose beads, washed, and eluted, and the cross-links were reversed. Purified DNA was amplified by PCR using primers flanking the predicted TRPS1-binding site within the EIF3H promoter (forward, 5′-GACCTCCCTCCAAAGTCACA-3′; reverse, 5′-ACGGACAGAGATGAGCACAA-3′) under the following conditions: initial denaturation at 95 °C for 5 min; 35 cycles of 95 °C for 30 s, 58 °C for 30 s, and 72 °C for 30 s; and a final extension at 72 °C for 5 min. The products were visualized by 2% agarose gel electrophoresis.

### 2.8. Protein Extraction and Western Blot (WB) Analysis

Protein extraction reagents were obtained from KeyGEN BioTECH Co., Ltd. (Nanjing, China), and total protein was extracted from TNBC cells using lysis buffer containing 1% phenylmethylsulfonyl fluoride (PMSF, Cat# RM70000, ABclonal, Wuhan, China) and protease inhibitor cocktail (Cat# RM02916, ABclonal, China). Bicinchoninic acid (BCA) assay was adopted to measure the protein concentration. Equal amounts of protein were loaded on a 10% sodium dodecyl sulfate-polyacrylamide gel electrophoresis (SDS-PAGE) and transferred to polyvinylidene difluoride (PVDF) membranes (Millipore, Burlington, MA, USA). Then, the blots were blocked in 5% skimmed milk with 0.1% Tween 20 at room temperature for 1 h, followed by incubation at 4 °C overnight with primary antibodies diluted in Universal Antibody Dilution Buffer (Cat# RM02955, ABclonal, China). The membranes were washed with tris-buffered saline with 0.1% Tween 20 (TBST) and then incubated with HRP-conjugated goat anti-rabbit IgG (H+L) secondary antibody (Cat# SA00001-2, 1:10,000, Proteintech, China) at room temperature for 2 h. The Western blot analysis involved the use of the following primary antibodies: anti-EIF3H (clone D9C1, Cat# 3413, 1:1000, Cell Signaling Technology, Danvers, MA, USA), anti-β-tubulin (polyclonal, Cat# 10094-1-AP, 1:2000, Proteintech, China), anti-TRPS1 (polyclonal, Cat# 21938-1-AP, 1:1000, Proteintech, China), anti-LDHA (polyclonal, Cat# 19987-1-AP, 1:2000, Proteintech, China), anti-MCT1 (polyclonal, Cat# 20139-1-AP, 1:2000, Proteintech, China), anti-phospho-STAT6 (Tyr641) (clone 250940C12, Cat# 86641-1-RR, 1:1000, Proteintech, China), anti-STAT6 (clone 4F19, Cat# 82630-1-RR, 1:2000, Proteintech, China), anti-HA tag (polyclonal, Cat# 51064-2-AP, 1:2000, Proteintech, China), and anti-ubiquitin (polyclonal, Cat# 10201-2-AP, 1:8000, Proteintech, China). The blots were detected using an enhanced chemiluminescence (ECL) kit (NcmECL Ultra, NCM Biotech, Suzhou, China).

### 2.9. CCK-8, Colony Formation, and EdU Incorporation Assays

Cell viability was monitored via the Cell Counting Kit-8 colorimetric assay (Beyotime Biotechnology, Shanghai, China; cat. no. C0043), which is based on the water-soluble tetrazolium salt (WST-8) method [38]. After seeding at 1 × 10^3^ cells per well in 96-well plates, cultures were sampled at three time points (24, 48, and 72 h); at each time point, wells received 10 μL of CCK-8 solution, and following a 2 h incubation at 37 °C, optical density at 450 nm was quantified on a microplate reader (Multiskan Sky, Thermo Fisher Scientific, USA).

For colony formation assays, cells were seeded in 6-well plates (1 × 10^3^ cells/well) and cultured in 10% FBS-supplemented medium for roughly two weeks. Colonies were fixed with 4% paraformaldehyde (20 min), stained with 0.1% crystal violet at room temperature, and counted.

EdU incorporation was assessed using an EdU assay kit (RiboBio, Guangzhou, China; cat. no. C10310). Cells seeded in 24-well plates (2 × 10^4^ cells/well) were allowed to attach overnight and then exposed to EdU reagent for 2 h at 37 °C per the manufacturer’s protocol. Following fixation and permeabilization, nuclei were counterstained with DAPI, and images from at least three random fields per sample were captured on a Leica fluorescence microscope to determine the percentage of EdU-positive cells.

### 2.10. Transwell Assay

A total of 2 × 10^4^ cells were planted in the upper layer of transwell membrane, and 10% fetal bovine serum was added to the lower chamber to encourage cell migration. The cells that traversed the membrane were stained with crystal violet for 20 min and examined using a microscope following 24 h incubation at 37 °C in a 5% CO_2_ environment.

### 2.11. In Vivo Mouse Models

All animal procedures were performed under approval no. IACUC-2310080 (granted 30 October 2023) from the Institutional Animal Care and Use Committee of the First Affiliated Hospital of Nanjing Medical University, following the NIH Guide for the Care and Use of Laboratory Animals; every effort was made to minimize animal suffering. Twenty 4-week-old female BALB/c nude mice were randomly assigned to four groups (*n* = 5/group) and received bilateral subcutaneous flank injections of the indicated stably transfected MDA-MB-231 cells. Tumor volume was tracked weekly (volume = π/6 × width^2^ × length), and after four weeks tumors were excised, weighed, and processed for further analysis.

### 2.12. Immunoprecipitation

Protease inhibitor-supplemented RIPA buffer (Beyotime) was used to lyse MDA-MB-231 cells, and the resulting lysates were subjected to protein quantification via a BCA assay kit (Beyotime), based on the bicinchoninic acid method [39]. Cell lysates were pre-cleared with Protein A/G-agarose beads (Beyotime) for 30 min at 4 °C, followed by immunoprecipitation with specific antibodies overnight at 4 °C. The next day, the samples were incubated with Protein A/G-agarose beads for an additional 3 h. The immunocomplexes were washed five times with RIPA buffer, and bound proteins were eluted by boiling in SDS-PAGE loading buffer for subsequent Western blot analysis.

### 2.13. Metabolic Assays

Glucose consumption, lactate production, and intracellular ATP levels were determined using the corresponding commercial assay kits (Beyotime Biotechnology, Shanghai, China) following the manufacturers’ protocols. The extracellular acidification rate (ECAR) was monitored in real time using a Seahorse XF Analyzer (Agilent Technologies, Santa Clara, CA, USA) with sequential injection of glucose, oligomycin, and 2-deoxy-D-glucose (2-DG). ECAR values were normalized to the total protein content of each well.

### 2.14. Immunoprecipitation Coupled with Mass Spectrometry (IP/MS)

Immunoprecipitation of MDA-MB-231 lysates was carried out as described above using a specific primary antibody and Protein A/G-agarose beads (Beyotime). The resulting immune complexes were resolved by SDS-PAGE, visualized with a silver staining kit (Beyotime, China), and subjected to mass spectrometry to identify differentially bound proteins.

### 2.15. Conditioned Medium (CM) Collection

Conditioned medium (CM) was prepared by culturing cells in serum-free medium following the indicated treatments. After 48 h of incubation, the supernatant was collected, centrifuged, and filtered through 0.22-μm membranes to obtain the CM.

### 2.16. Molecular Docking and Structural Simulation

The three-dimensional structures of EIF3H and LDHA were predicted using AlphaFold3. Protein–protein docking was performed using the HDOCK server, and the binding interface and interacting residues were analyzed and visualized using PyMOL v2.5.4 (Schrödinger, New York, NY, USA).

### 2.17. Statistical Analysis

Statistical analyses were performed using SPSS 25.0 (IBM, USA) and GraphPad Prism 11.0.0 (GraphPad Software, Boston, MA, USA), with each experiment repeated independently at least three times and data expressed as mean ± *SD* unless stated otherwise. Two-group comparisons used the unpaired two-tailed Student’s t-test, while comparisons among three or more groups against a single control used one-way ANOVA with Dunnett’s multiple comparisons test. Survival analyses were performed using the Kaplan–Meier method with the log-rank test, and Cox proportional hazards regression was used for survival-related analyses. Correlations between gene expression levels were evaluated using Pearson’s correlation analysis. A two-tailed *p* < 0.05 was considered statistically significant.

## 3. Results

### 3.1. Upregulated EIF3H Expression Correlates with Poor Prognosis in TNBC Patients

**Figure 1:**
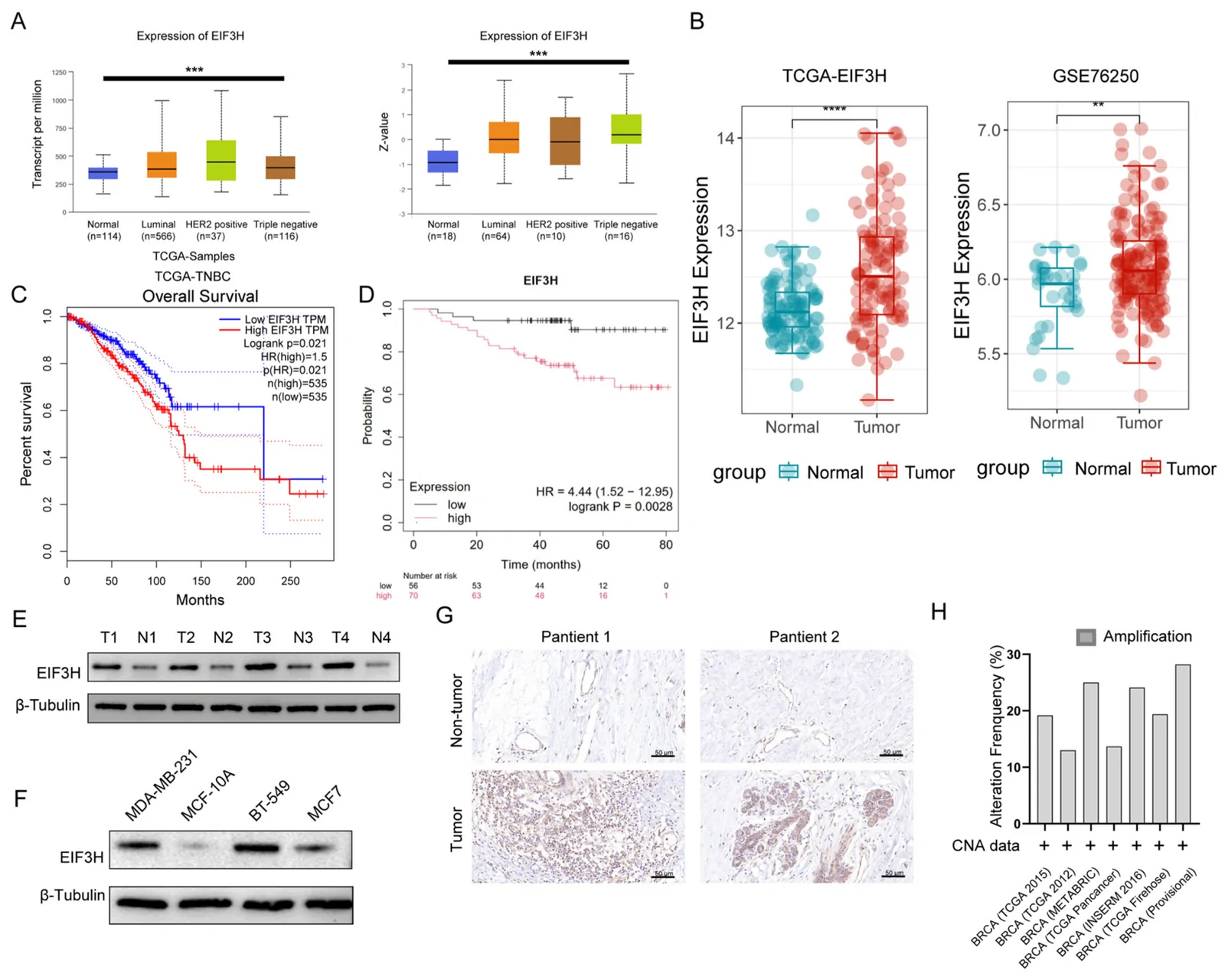
Upregulated EIF3H Expression Correlates with Poor Prognosis in TNBC Patients. (**A**) EIF3H mRNA expression across breast cancer molecular subtypes in the TCGA-BRCA dataset (left; normal, *n* = 114; luminal, *n* = 566; HER2-positive, *n* = 37; triple-negative, *n* = 116) and EIF3H protein expression across molecular subtypes in the CPTAC breast cancer cohort (right; normal, *n* = 18; luminal, *n* = 64; HER2-positive, *n* = 10; triple-negative, *n* = 16). (**B**) EIF3H mRNA expression in Normal versus TNBC tissues from the TCGA-TNBC cohort (left) and the GSE76250 dataset (right). (**C**) Overall survival analysis of TNBC patients stratified by EIF3H expression in the TCGA-TNBC cohort. Patients were divided into high- and low-expression groups based on the median EIF3H TPM value. (log-rank *p* = 0.021; *HR* = 1.5, 95% *CI*: 1.06–2.12). (**D**) Overall survival analysis of TNBC patients stratified by EIF3H expression using the Kaplan–Meier plotter database. (log-rank *p* = 0.0028; *HR* = 4.44, 95% *CI*: 1.52–12.95). (**E**) Western blot analysis of EIF3H protein expression in paired TNBC tumor (T) and adjacent non-tumor (N) tissues from four representative patients. β-Tubulin served as the loading control. (**F**) Western blot analysis of EIF3H protein levels in normal breast epithelial cells (MCF-10A), non-TNBC breast cancer cells (MCF7), and TNBC cell lines (MDA-MB-231, BT-549). β-Tubulin served as the loading control. (**G**) Representative immunohistochemical staining of EIF3H in paired TNBC tumor and adjacent normal tissues. Scale bar, 50 μm (×400 magnification). (**H**) Copy number alteration (CNA) frequency of EIF3H across breast cancer datasets from cBioPortal, including three independent cohorts (TCGA, METABRIC, and INSERM 2016). Box plots show the median (center line), interquartile range (box), and minimum–maximum (whiskers). The original uncropped Western blot image for panel (**E**,**F**) is shown in Supplementary Figure S6 (Original Images 44–47). ** *p* < 0.01, *** *p* < 0.001, **** *p* < 0.0001.

To examine EIF3H’s role in TNBC progression, we mined transcriptomic data from TCGA and GEO alongside proteomic data from CPTAC. EIF3H was consistently upregulated in TNBC relative to normal breast tissue at both mRNA and protein levels (Figure 1A,B), and survival analysis in the TCGA-BRCA cohort and Kaplan–Meier plotter database showed that higher EIF3H expression correlated with poorer overall survival among TNBC patients (Figure 1C,D).

In addition, EIF3H mRNA and protein levels were examined in TNBC tissues and matched non-tumor samples from our patient cohort, showing results consistent with those derived from public datasets. Western blot and immunohistochemistry (IHC) further validated EIF3H upregulation, revealing markedly higher EIF3H protein levels in TNBC carcinoma tissues than in adjacent normal tissues. EIF3H protein expression was also higher in TNBC cell lines (MDA-MB-231 and BT-549) than in normal breast cell lines (MCF-10A) and the non-TNBC breast cancer cell line MCF7 (Figure 1E–G). Moreover, analysis of seven breast cancer datasets from cBioPortal revealed frequent EIF3H amplification, with alteration frequencies ranging from 13% to 28% across all seven datasets (Figure 1H). Collectively, these findings demonstrate that EIF3H is frequently upregulated in TNBC and is associated with adverse patient outcomes.

### 3.2. TRPS1 Transcriptionally Regulates EIF3H

**Figure 2:**
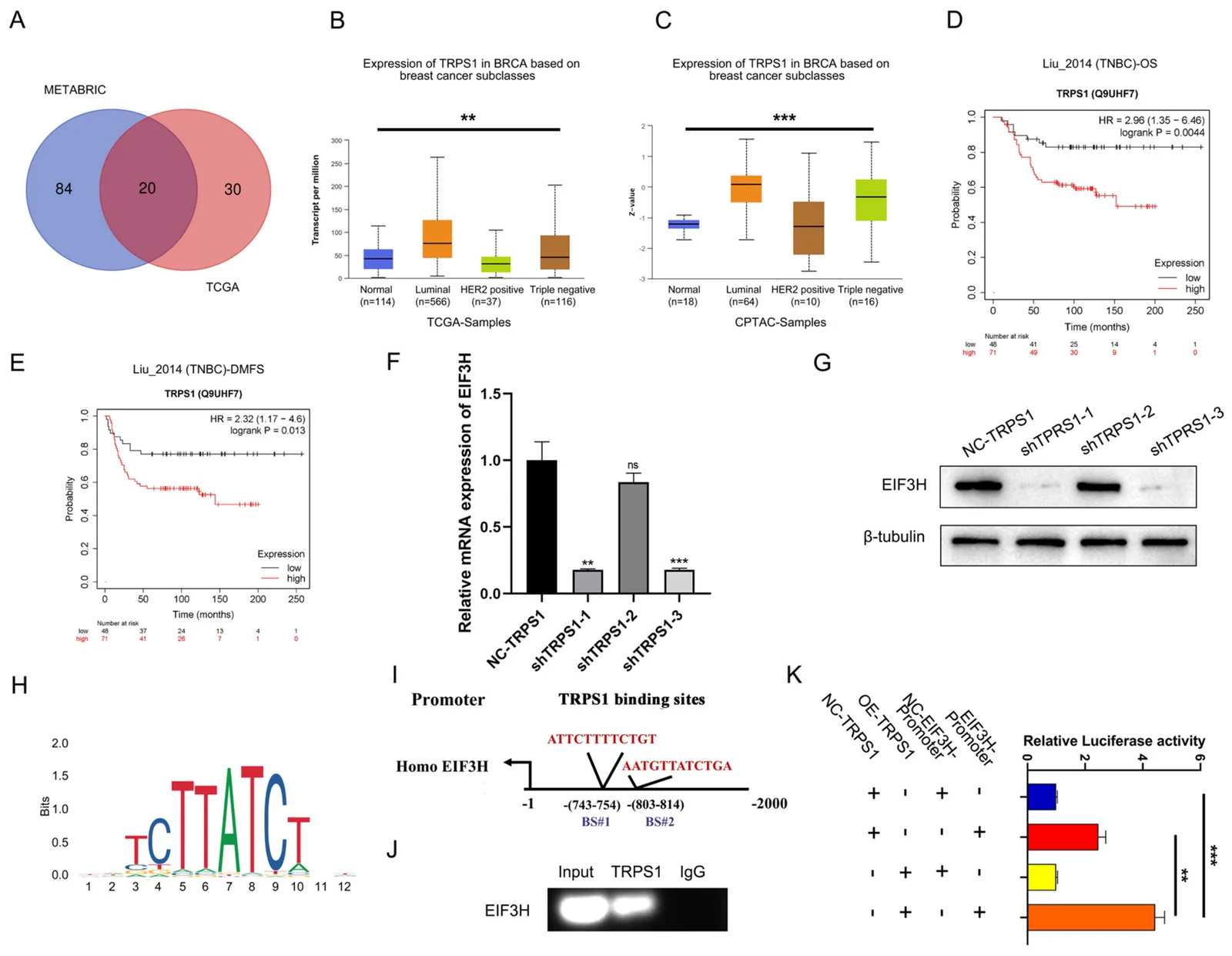
EIF3H is transcriptionally regulated by TRPS1. (**A**) Overlapping analysis of EIF3H co-expressed genes identified from the TCGA-BRCA and METABRIC datasets. (**B**) TRPS1 mRNA expression levels across breast cancer subtypes in TCGA database. (**C**) TRPS1 protein expression levels across breast cancer subtypes in CPTAC database. (**D**,**E**) Kaplan–Meier analysis of overall survival (**D**) and distant metastasis-free survival (**E**) according to TRPS1 protein expression in the Liu_2014 triple-negative breast cancer proteomics cohort (Kaplan–Meier plotter; (**D**): log-rank *p* = 0.0044, *HR* = 2.96, 95% *CI*: 1.35–6.46; (**E**): log-rank *p* = 0.013, *HR* = 2.32, 95% *CI*: 1.17–4.6). (**F**) EIF3H mRNA expression in MDA-MB-231 cells following TRPS1 knockdown with three independent shRNAs, assessed by qRT-PCR. (**G**) EIF3H protein expression in MDA-MB-231 cells following TRPS1 knockdown, assessed by Western blotting. (**H**) The binding motif of TRPS1 obtained from the JASPAR database. (**I**) Putative TRPS1-binding sites within the genomic sequence upstream of the transcription start site (TSS) of the EIF3H gene. (**J**) ChIP assay showing TRPS1 occupancy at the EIF3H promoter; PCR products were resolved by agarose gel electrophoresis. (**K**) Luciferase reporter assay of EIF3H promoter activity in MDA-MB-231 cells co-transfected with TRPS1 overexpression or control vectors. The original uncropped Western blot images for panel G are shown in Supplementary Figure S6 (Original Images 1–2). Significance was assessed using one-way ANOVA with Dunnett’s multiple comparisons test ((**F**); each shRNA vs. shNC) or Tukey’s multiple comparisons test (**K**); ** *p* < 0.01, *** *p* < 0.001; ns, not significant.

Transcription factors (TFs) play critical roles in regulating gene expression. To identify upstream TFs potentially controlling EIF3H, we first used the UALCAN website to screen for genes significantly correlated with EIF3H expression [40] (Pearson *r* ≥ 0.3) in the TCGA-BRCA dataset, yielding 50 genes (Supplementary Table S2). A parallel analysis was performed for the METABRIC breast cancer cohort using the cBioPortal database (Spearman *r* ≥ 0.3), yielding 98 genes (Supplementary Table S3). Intersection of the two datasets yielded 20 overlapping genes (Supplementary Table S4). Given that UALCAN and cBioPortal rank co-expressed genes without functional annotation, the 20 overlapping genes were further cross-referenced against the curated catalog of human transcription factors. TRPS1 was the only transcription factor among the overlapping genes and therefore emerged as the candidate regulator of EIF3H expression (Figure 2A). UALCAN analysis further confirmed that TRPS1 was significantly upregulated in breast cancer samples at both the mRNA level in the TCGA cohort and the protein level in the CPTAC cohort (Figure 2B,C). Moreover, elevated TRPS1 protein expression was associated with poorer overall survival (OS) and distant metastasis-free survival (DMFS) in patients with TNBC (Figure 2D,E). A positive correlation between TRPS1 and EIF3H expression was observed in the GSE65194, GSE76250, METABRIC, and TCGA-TNBC cohorts (Supplementary Figure S1A–D). Additionally, analysis of the GSE65194 dataset demonstrated that TRPS1 mRNA expression was significantly elevated in TNBC tissues compared with normal breast tissues (Supplementary Figure S1E), and this finding was corroborated in the GSE76250 dataset, which showed higher TRPS1 expression in tumor tissues relative to adjacent non-tumoral tissues (Supplementary Figure S1F). Increased TRPS1 expression in breast cancer samples compared with normal breast tissues was also observed in additional datasets (Supplementary Figure S1G).

To validate the TRPS1–EIF3H relationship, we silenced TRPS1 in the TNBC cell line MDA-MB-231 using three independent short hairpin RNAs (shTRPS1-1, shTRPS1-2, and shTRPS1-3), and the knockdown efficiency of each shRNA was verified at both the mRNA and protein levels (Supplementary Figure S1H,I). TRPS1 depletion reduced EIF3H expression at both the mRNA and protein levels (Figure 2F,G). Notably, shTRPS1-2, which failed to efficiently deplete TRPS1, did not significantly affect EIF3H expression, whereas shTRPS1-1 and shTRPS1-3 markedly suppressed EIF3H, indicating that the reduction in EIF3H paralleled TRPS1 knockdown efficiency (Figure 2F,G and Supplementary Figure S1H,I). For subsequent functional studies, shTRPS1-1 was selected owing to its robust and consistent knockdown efficiency. Based on the TRPS1 binding motif predicted by the JASPAR database, we identified putative TRPS1 binding sites within the EIF3H promoter region (Figure 2H,I), and ChIP analysis confirmed TRPS1 occupancy at the EIF3H promoter (Figure 2J). A dual-luciferase reporter assay further showed that TRPS1 overexpression significantly enhanced EIF3H promoter activity (Figure 2K). Collectively, these results demonstrate that TRPS1 transcriptionally regulates EIF3H expression.

### 3.3. EIF3H Exerts Pro-Tumorigenic Effects in TNBC Cells in Vitro and in Vivo

**Figure 3:**
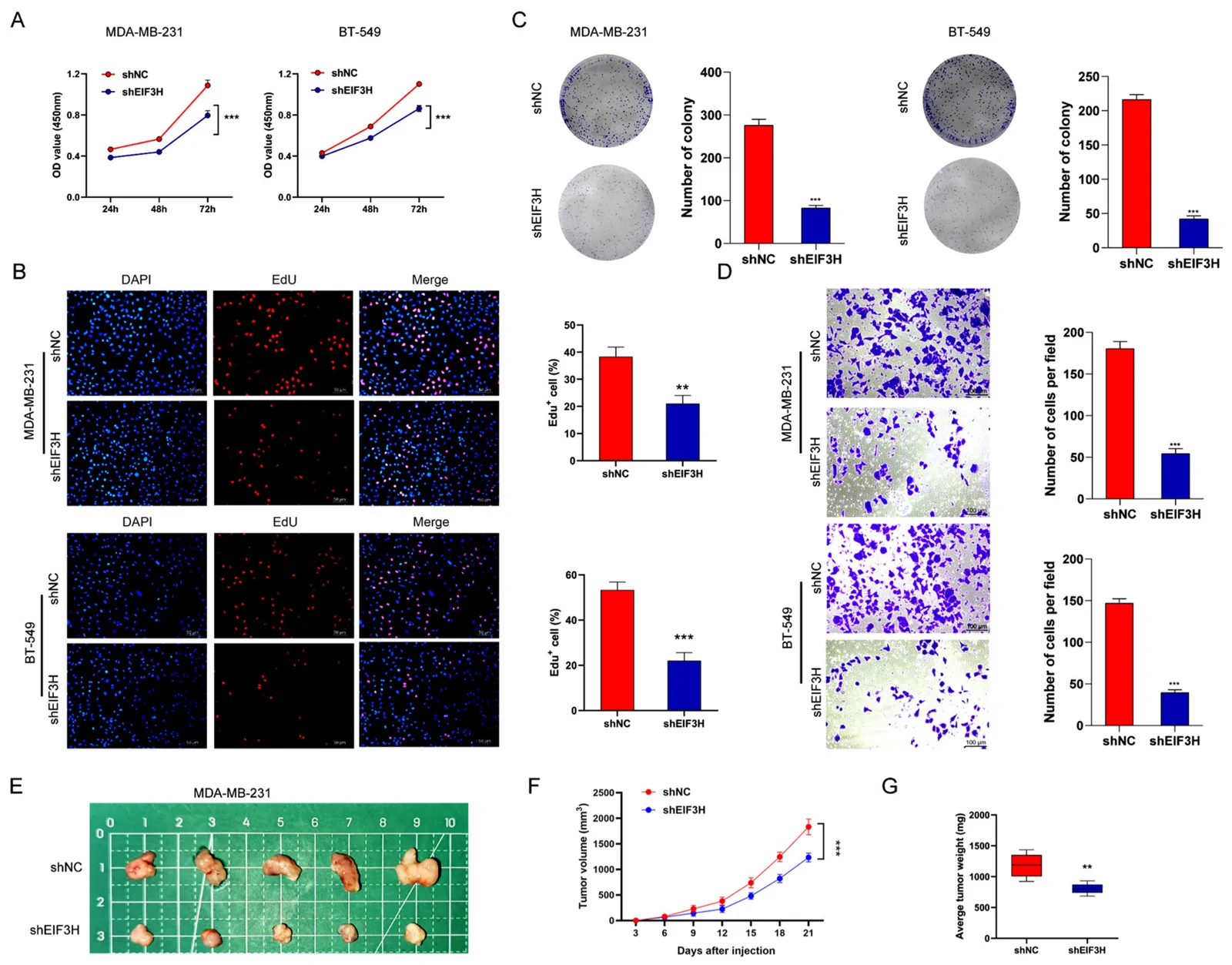
EIF3H exerts pro-tumorigenic effects in TNBC cells in vitro and in vivo (**A**) CCK8 assay of MDA-MB-231 and BT-549 cells with lentiviral knockdown of EIF3H (shEIF3H, i.e., shEIF3H-2) or negative control (shNC). (**B**) 5-ethynyl-2′-deoxyuridine (EdU) staining assays of MDA-MB-231 and BT-549 cells with lentiviral knockdown of EIF3H (shEIF3H) or negative control (shNC). Scale bar = 50 μm. (**C**) Colony formation assay of MDA-MB-231 and BT-549 cells with lentiviral knockdown of *EIF3H* or negative control. (**D**) Migration of MDA-MB-231 and BT-549 cells with *EIF3H* knockdown, assessed by transwell assay. Scale bar = 100 μm. (**E**). Representative subcutaneous tumors collected from BALB/c nude mice. (**F**) Tumor growth curves of subcutaneous xenografts in MDA-MB-231-bearing BALB/c nude mice. (**G**) Tumor weight of subcutaneous xenografts in MDA-MB-231-bearing BALB/c nude mice. Statistical significance was determined by unpaired two-tailed Student’s t-test; for (**A**,**F**), values at the final time point were compared; ** *p* < 0.01, *** *p* < 0.001.

Prior to functional characterization, we first confirmed the efficiency of lentivirus-mediated EIF3H overexpression and knockdown in MDA-MB-231 and BT-549 cells at both the protein and mRNA levels. Western blot analysis demonstrated that EIF3H protein levels were markedly elevated in cells transduced with OE-EIF3H lentivirus compared with negative control (OE-NC) cells. Among the three independent shRNAs targeting EIF3H, shEIF3H-2 exhibited the highest knockdown efficiency in both cell lines and was therefore used for all subsequent knockdown experiments (hereafter referred to as shEIF3H) (Supplementary Figure S5A,B). Similarly, LDHA knockdown efficiency was validated in the rescue experiment setting, and GPR65 knockdown efficiency was confirmed in THP-1-derived macrophages (Supplementary Figure S4C–F). These results confirmed the successful establishment of stable cell lines for subsequent functional assays. CCK-8 and EdU incorporation assays showed that EIF3H overexpression enhanced TNBC cell proliferation, whereas EIF3H knockdown inhibited it (Figure 3A,B; Supplementary Figure S2A,B). Similarly, colony formation assays demonstrated that EIF3H increased the clonogenic potential of both cell lines (Figure 3C; Supplementary Figure S2C). Transwell migration assays further revealed that EIF3H overexpression promoted migratory capacity, while EIF3H depletion impaired cell motility (Figure 3D; Supplementary Figure S2D). Together, these in vitro results indicate that EIF3H promotes proliferation, clonogenicity, and migration in TNBC cells.

We next established xenograft models in nude mice. Tumors in the shEIF3H group were significantly smaller, exhibited slower growth, and had lower final tumor weights than those in the shNC group (Figure 3E–G). Conversely, OE-EIF3H tumors showed enhanced tumor growth (Supplementary Figure S2E–G). These in vivo results corroborated the in vitro findings, confirming that EIF3H promotes TNBC tumorigenesis.

### 3.4. LDHA Is Identified as a Binding Partner of EIF3H

**Figure 4:**
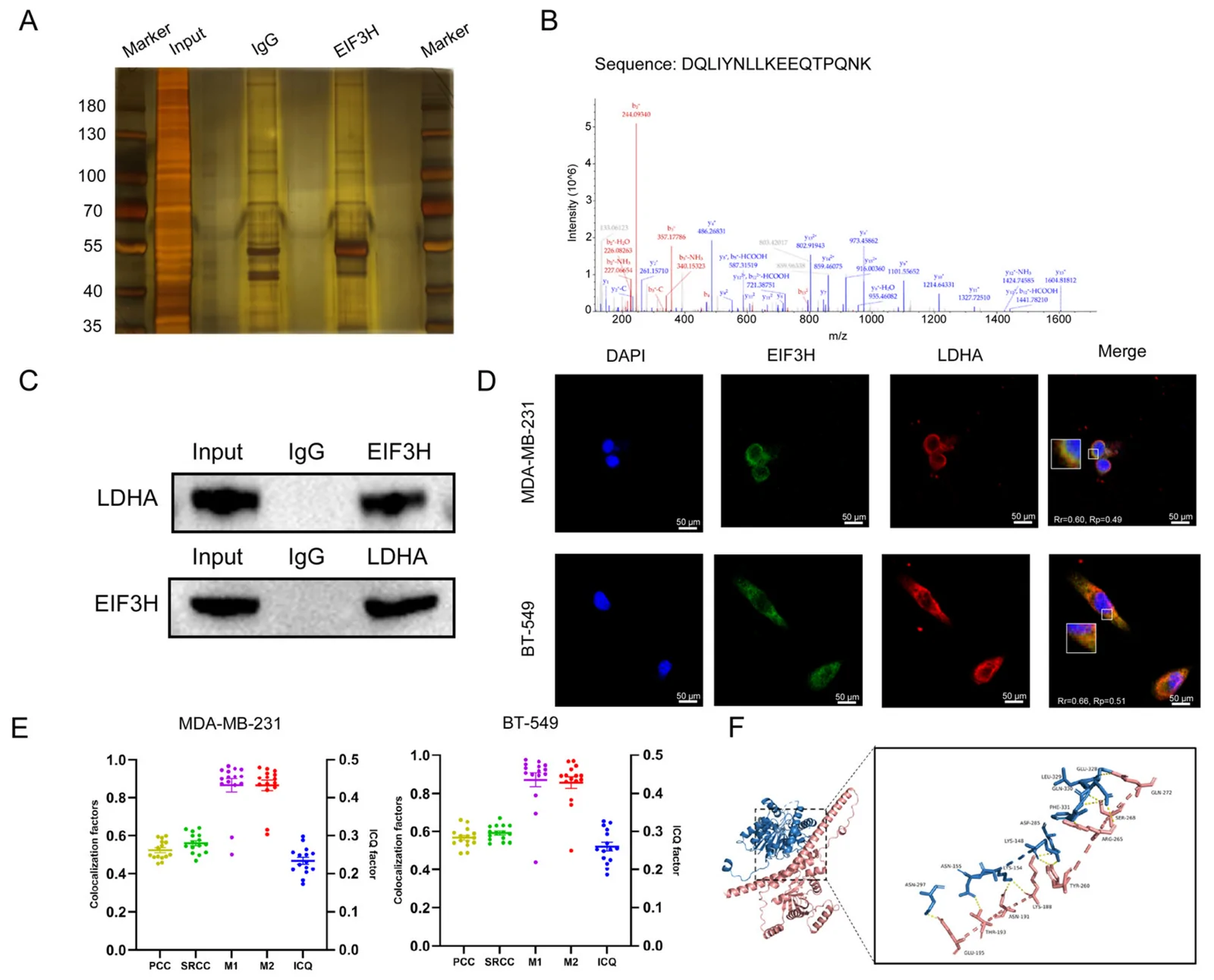
Identification of LDHA as a binding partner of EIF3H. (**A**) Silver staining assay used to Identify the EIF3H-binding proteins in MDA-MB-231 cells. (**B**) Peptide sequences of LDHA identified within the Co-IP complex by mass spectrometry. (**C**) Western blotting of the Co-IP complex confirming the EIF3H–LDHA interaction in MDA-MB-231 cells. (**D**) Representative immunofluorescence images showing the colocalization of EIF3H (green) and LDHA (red) in MDA-MB-231 and BT-549 cells; nuclei were counterstained with DAPI (blue). Insets show magnified views of the boxed regions, highlighting the cytoplasmic colocalization of EIF3H and LDHA. Rr and Rp indicate Pearson’s correlation coefficients without and with Costes’ threshold, respectively, calculated for the representative cell shown. Scale bar, 50 μm. (**E**) Quantitative colocalization analysis of EIF3H and LDHA in MDA-MB-231 and BT-549 cells (*n* = 15 cells per cell line). PCC, SRCC, M1 and M2 are plotted against the left Y axis; ICQ is plotted against the right Y axis. Data are presented as mean ± *SEM*. (**F**) The three-dimensional structure of the EIF3H–LDHA complex was simulated by molecular docking. The original uncropped Western blot images for panel (**C**) are shown in Supplementary Figure S6 (Original Images 3–4).

To elucidate the mechanism by which EIF3H drives TNBC progression, we performed silver staining and mass spectrometry to identify proteins co-precipitating with EIF3H. Several differential protein bands were observed between IgG and EIF3H immunoprecipitates (Figure 4A). Because metabolic reprogramming—particularly enhanced glycolysis—is a hallmark of cancer, we focused on glycolytic enzymes among the candidate proteins. Lactate dehydrogenase A (LDHA) was selected for further validation due to its central role in glycolysis (Figure 4B).

Co-immunoprecipitation (Co-IP) confirmed the interaction between EIF3H and LDHA in MDA-MB-231 cells (Figure 4C). Immunofluorescence assays further showed pronounced co-localization of EIF3H and LDHA in both MDA-MB-231 and BT-549 cells (Figure 4D). Quantitative colocalization analysis confirmed substantial overlap between the two proteins. In MDA-MB-231 cells, the Pearson’s correlation coefficient (PCC) was 0.524 ± 0.013, Spearman’s rank correlation coefficient (SRCC) was 0.561 ± 0.013, Manders’ overlap coefficients M1 and M2 were 0.864 ± 0.035 and 0.864 ± 0.027, and the intensity correlation quotient (ICQ) was +0.234 ± 0.009 (mean ± *SEM*, *n* = 15 cells). Consistent results were obtained in BT-549 cells, with a PCC of 0.568 ± 0.013, SRCC of 0.592 ± 0.010, M1 and M2 of 0.871 ± 0.036 and 0.857 ± 0.030, and ICQ of +0.260 ± 0.011 (Figure 4E). Structural simulation and molecular docking predicted that residues ASN297, ASP285, PHE331, LEU329, and GLU328 within the C-terminal domain of LDHA interact with GLU195, LYS188, ARG265, SER268, and GLN272 in EIF3H (Figure 4F).

### 3.5. EIF3H Regulates LDHA Ubiquitination and Protein Stability

**Figure 5:**
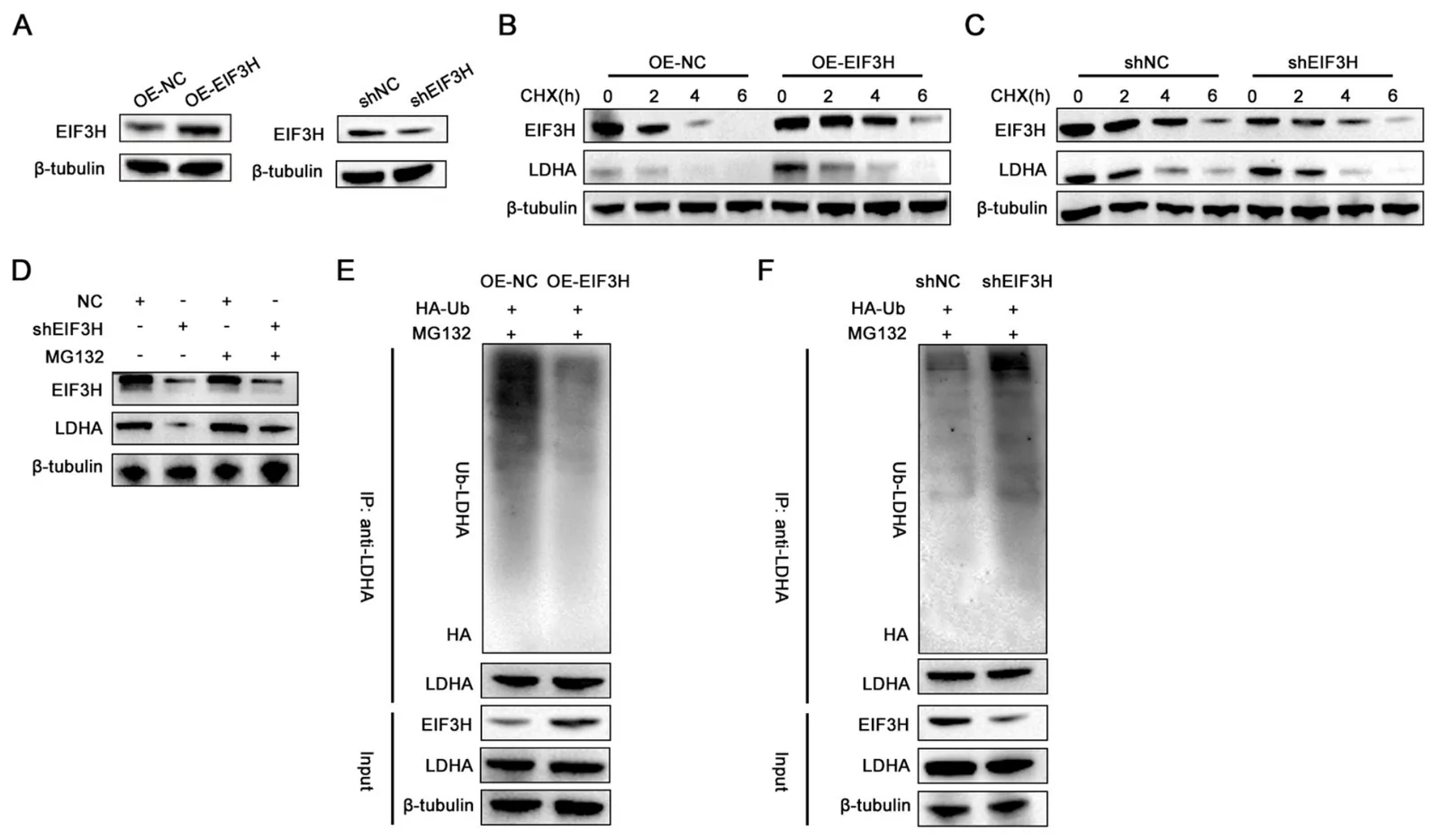
EIF3H regulates LDHA ubiquitination and protein stability. (**A**) Western blotting of EIF3H expression in EIF3H-overexpressing or vector control and EIF3H-knockdown or negative control MDA-MB-231 cells. (**B**) Time-course analysis of EIF3H and LDHA protein levels in EIF3H-overexpressing or vector MDA-MB-231 treated with cycloheximide (CHX). (**C**) Time-course analysis of EIF3H and LDHA protein levels in EIF3H-knockdown or negative control MDA-MB-231 treated with cycloheximide. (**D**) EIF3H and LDHA protein levels in EIF3H-knockdown or negative control MDA-MB-231 treated with proteasome inhibitor, MG132. (**E**) Ubiquitination of LDHA in EIF3H-overexpressing or vector control MDA-MB-231 cells co-transfected with HA-tagged ubiquitin and treated with MG132. LDHA was immunoprecipitated, and ubiquitin-conjugated LDHA was detected by immunoblotting using an anti-HA antibody. (**F**) Ubiquitination of LDHA in EIF3H-knockdown or negative control MDA-MB-231 cells, analyzed as in (**E**). The original uncropped Western blot images for panels (**A**–**F**) are shown in Supplementary Figure S6 (Original Images 5–27).

As EIF3H contains a JAMM/MPN domain characteristic of metalloprotease deubiquitylases, we next examined whether EIF3H affects the ubiquitination and protein stability of LDHA. To test this hypothesis, MDA-MB-231 and BT-549 cells were treated with the protein synthesis inhibitor cycloheximide (CHX). EIF3H overexpression markedly attenuated LDHA degradation, whereas LDHA degradation was significantly accelerated in EIF3H-knockdown cells (Figure 5A–C). Consistently, shEIF3H or control (Ctrl) MDA-MB-231 cells were treated with the proteasome inhibitor MG132, which substantially restored LDHA protein levels (Figure 5D). These results indicate that EIF3H regulates LDHA stability via a proteasome-dependent pathway.

We then investigated whether EIF3H modulates LDHA proteolysis through the ubiquitin–proteasome system (UPS). TNBC cells overexpressing EIF3H or with EIF3H knockdown were transiently co-transfected with plasmids encoding HA-tagged ubiquitin. The results showed that EIF3H-overexpressing MDA-MB-231 cells exhibited significantly reduced polyubiquitination of LDHA compared with NC cells, whereas EIF3H-knockout MDA-MB-231 cells displayed dramatically increased polyubiquitination compared with shNC cells (Figure 5E,F). Taken together, these findings suggest that EIF3H restrains LDHA polyubiquitination and thereby inhibits proteasomal degradation. Whether EIF3H directly deubiquitinates LDHA warrants further investigation using in vitro deubiquitination assays.

### 3.6. LDHA Mediates the Pro-Tumorigenic Effects of EIF3H in TNBC Cells

**Figure 6:**
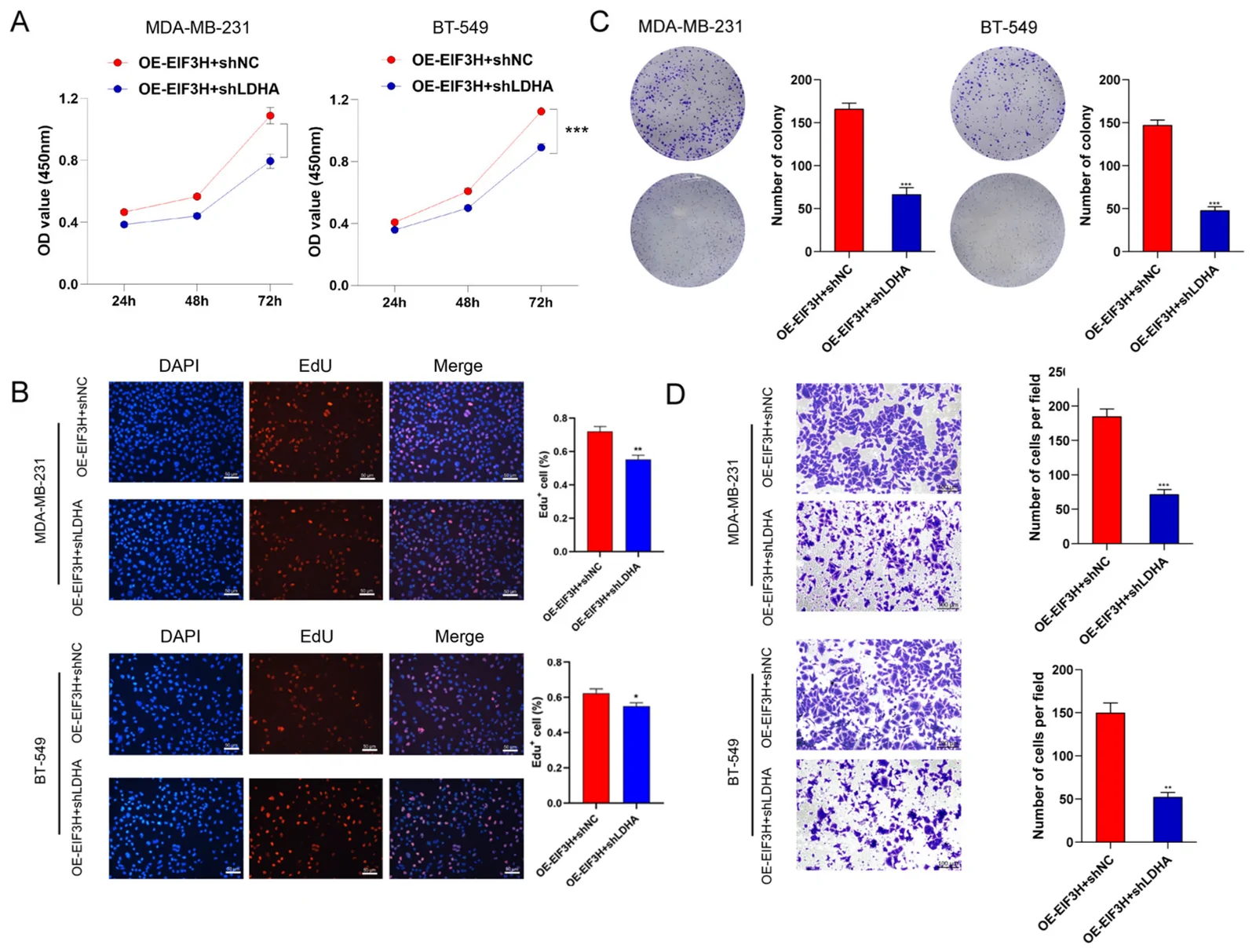
EIF3H promotes tumor progression via LDHA. MDA-MB-231 and BT-549 cells with stable EIF3H overexpression were further transduced with LDHA-targeting shRNA (shLDHA) or negative-control shRNA (shNC). (**A**) Proliferation assessed by CCK-8 assay. (**B**) EdU staining assay. Scale bar = 50 μm. (**C**) Colony formation assay. (**D**) Transwell migration assay. Scale bar = 100 μm. Significance was determined by unpaired two-tailed Student’s t-test; * *p* < 0.05, ** *p* < 0.01, *** *p* < 0.001.

To determine whether EIF3H promotes TNBC progression by regulating LDHA protein stability, we performed rescue experiments. LDHA was knocked down via lentiviral infection in MDA-MB-231 and BT-549 cells stably overexpressing EIF3H, and the knockdown efficiency of LDHA was validated at both the protein and mRNA levels (Supplementary Figure S5C,E). CCK-8 and EdU assays showed that LDHA knockdown markedly reversed the pro-proliferative effects of EIF3H overexpression in both cell lines (Figure 6A,B). Likewise, the enhanced colony formation induced by EIF3H overexpression was fully abolished by LDHA depletion (Figure 6C). In addition, transwell migration assays confirmed that LDHA knockdown eliminated the pro-migratory effect of EIF3H overexpression (Figure 6D).

Conversely, LDHA was re-introduced into EIF3H-knockdown MDA-MB-231 and BT-549 cells. LDHA restoration significantly rescued the impaired proliferation of EIF3H-depleted cells, as evidenced by CCK-8 and EdU assays (Supplementary Figure S3A,B), and likewise restored their migratory capacity in transwell assays (Supplementary Figure S3C).

Collectively, these bidirectional rescue experiments demonstrate that EIF3H promotes TNBC cell proliferation and migration through LDHA.

### 3.7. EIF3H Promote Glycolysis to Mediates Tumor Cell-Derived Lactate Production

Because of the Warburg effect, elevated lactate metabolism—and consequent acidification of the tumor microenvironment (TME)—is a common feature of solid tumors [39,41,42]. In TNBC specifically, LDHA drives lactate production while its export relies chiefly on monocarboxylate transporter 1 (MCT1, encoded by SLC16A1) [43,44].

We next assessed lactate metabolism in TNBC using bulk RNA-seq data from TCGA, which showed higher LDHA and MCT1 mRNA levels in tumor versus normal tissue (Supplementary Figure S4A,B). Kaplan–Meier analysis linked elevated LDHA expression to a trend toward worse overall survival in TNBC patients (Supplementary Figure S4C), with a similar pattern for SLC16A1 (Supplementary Figure S4D). Western blotting of clinical specimens corroborated these findings, showing increased LDHA and MCT1 protein in TNBC relative to adjacent normal tissue.

**Figure 7:**
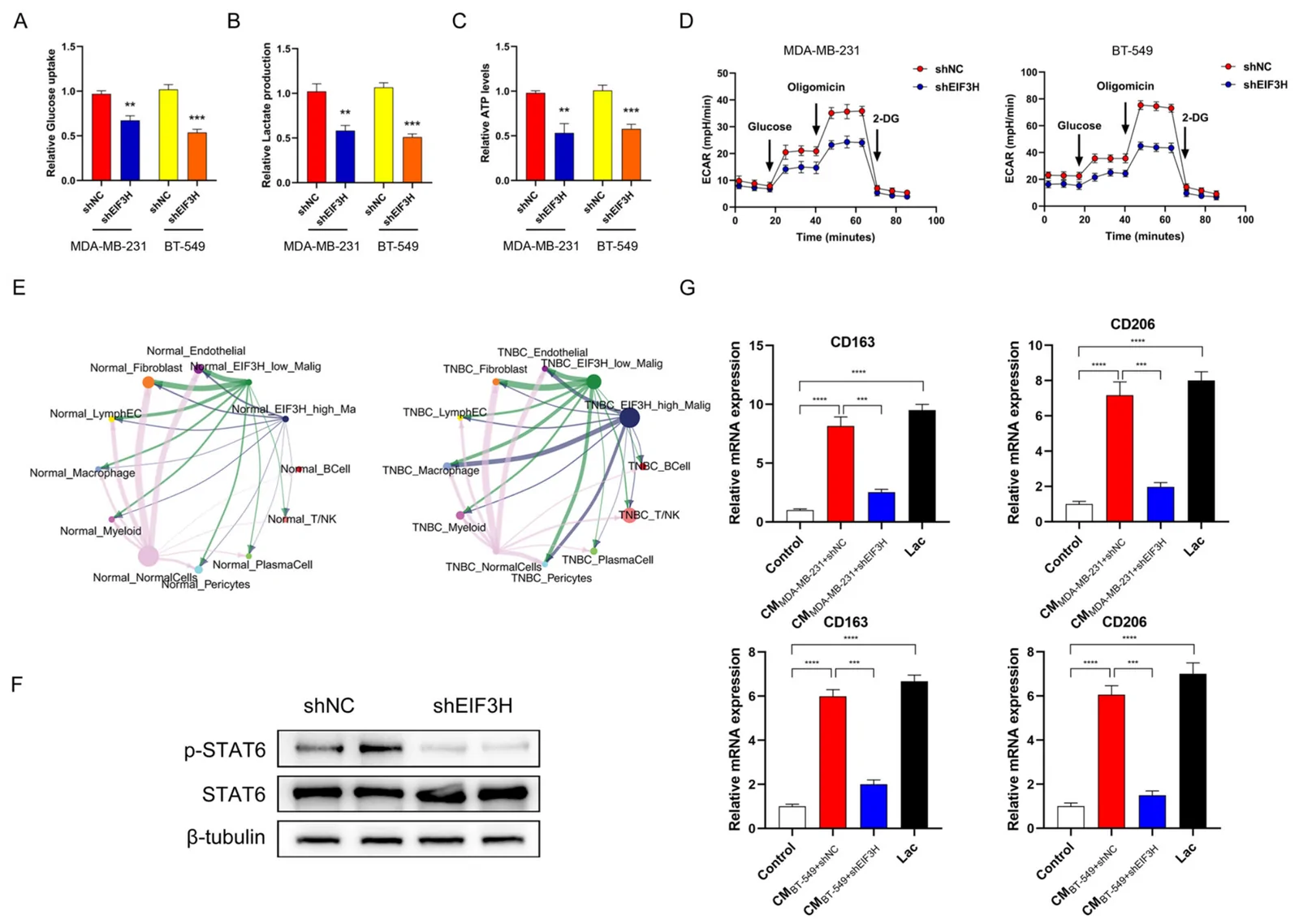
EIF3H promotes glycolysis and tumor cell-derived lactate production and induces M2 polarization of tumor-associated macrophages. MDA-MB-231 and BT-549 cells were transduced with EIF3H-targeting shRNA or negative control (shNC). (**A**) Glucose uptake. (**B**) Lactate production. (**C**) Intracellular ATP levels. (**D**) Real-time extracellular acidification rate (ECAR) profiles recorded after sequential injection of glucose (Glc), oligomycin (O), and 2-DG; quantification of glycolysis, glycolytic capacity, and glycolytic reserve is provided in Supplementary Figure S4E,F. (**E**) Chord diagrams showing the cell-cell interactions between major cell types in tumor (**right**) and normal (**left**) tissues. (**F**) p-STAT6 and STAT6 protein expression detected by Western blot in THP-1 cells treated with conditioned medium from EIF3H-knockdown or control MDA-MB-231 cells. (**G**) CD206 and CD163 mRNA expression detected by RT-qPCR in THP-1 cells treated with conditioned medium from EIF3H-knockdown or control MDA-MB-231 and BT-549 cells or with lactate (Lac). Significance was determined by unpaired two-tailed Student’s t-test (**A**–**C**) or one-way ANOVA with Tukey’s multiple comparisons test (**G**); ** *p* < 0.01, *** *p* < 0.001, **** *p* <0.0001.

We further validated the regulatory role of EIF3H in glycolysis by measuring glucose uptake, lactate production, intracellular ATP levels, and the extracellular acidification rate (ECAR) via Seahorse analysis (Figure 7A–D). Quantification of the ECAR profiles showed that EIF3H knockdown significantly reduced glycolysis, glycolytic capacity, and glycolytic reserve in both MDA-MB-231 and BT-549 cells (Supplementary Figure S4E,F). Overall, EIF3H enhanced both lactate production and lactate efflux in TNBC cells.

### 3.8. EIF3H-Driven Lactate Induces Macrophage M2 Polarization via GPR65

To assess how EIF3H affects interactions between TNBC cells and other components of the tumor microenvironment, we performed CellChat analysis (Figure 7E) using the TNBC single-cell dataset GSE161529. The results indicated that TNBC cells interact more strongly with macrophages than normal cells do; notably, EIF3H-positive TNBC cells showed particularly robust macrophage interactions. Treatment of THP-1–derived macrophages with conditioned medium (CM) from control MDA-MB-231 cells induced phosphorylation of STAT6, a key factor involved in M2 polarization, whereas this effect was markedly attenuated by CM from EIF3H-knockdown cells (Figure 7F). Moreover, CM from both MDA-MB-231 and BT-549 cells significantly increased the expression of the M2 macrophage markers CD163 and CD206 compared with the blank control; this induction was attenuated when the CM was derived from EIF3H-knockdown cells and was mimicked by exogenous lactate (Figure 7G).

**Figure 8:**
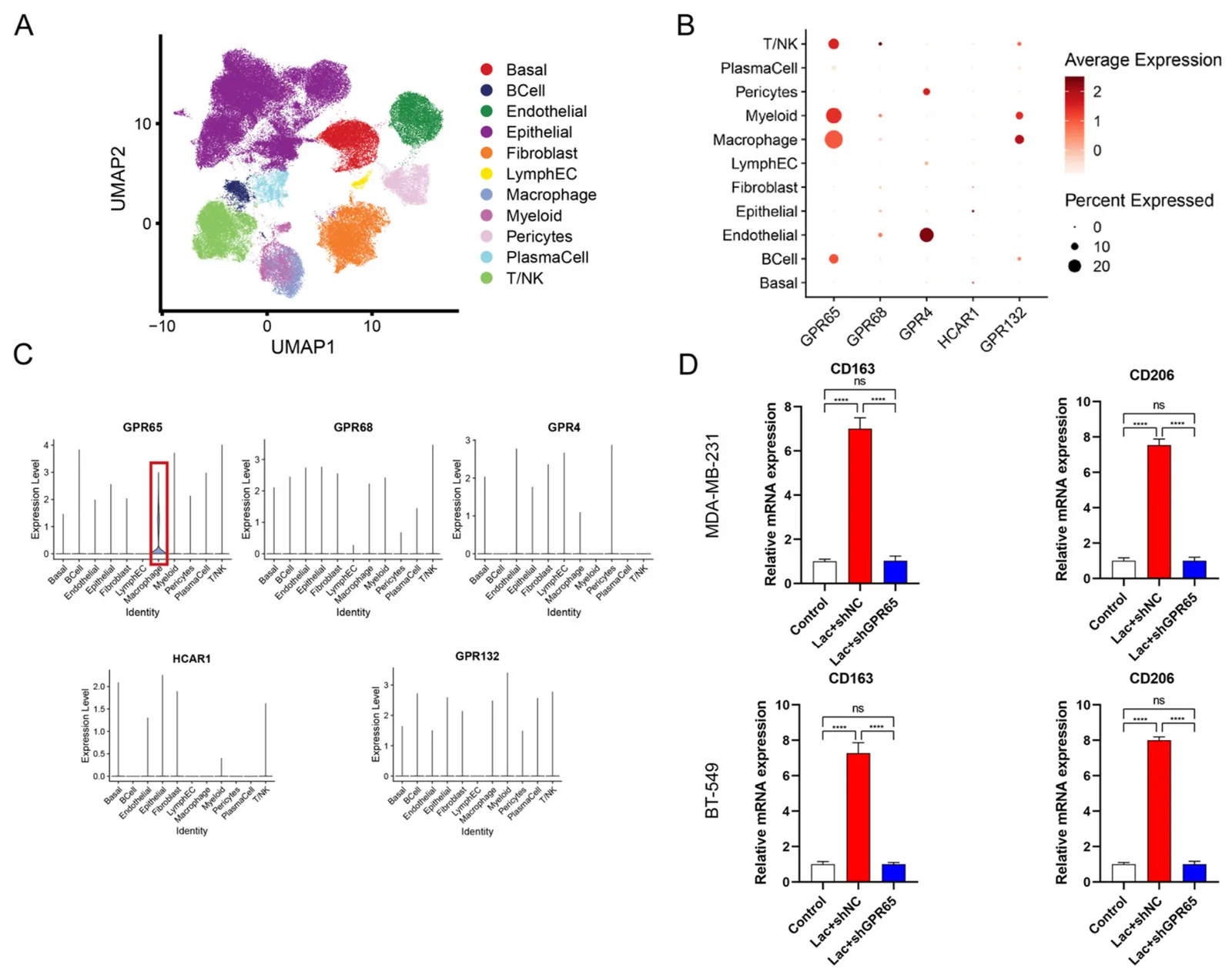
Lactate induces macrophage M2 polarization via GPR65. (**A**) Uniform manifold approximation and projection (UMAP) plot showing the major cell types. (**B**) Dot plot showing the expression of lactate receptors (GPR65, GPR68, GPR4, HCAR1, and GPR132) across major cell types. (**C**) Violin plot showing the expression of GPR65, GPR68, GPR4, HCAR1 and GPR132. The red box highlights macrophage-enriched GPR65 expression. (**D**) CD163 and CD206 mRNA expression in THP-1–derived macrophages transduced with shGPR65 or negative control (shNC) and treated with lactate, detected by RT-qPCR; upper panels: MDA-MB-231 system; lower panels: BT-549 system. Statistical significance was determined by one-way ANOVA followed by Tukey’s multiple comparisons test for (**D**); **** *p* < 0.0001; ns, not significant.

In the tumor microenvironment, lactate stimulation triggers signaling pathways mainly through lactate receptors, including HCAR1 (GPR81) and proton-sensing G-protein-coupled receptors (GPRs), such as GPR4, TDAG8 (GPR65), OGR1 (GPR68), and G2A (GPR132) [45,46,47]. These receptors exhibit distinct expression patterns and activate different signaling cascades depending on the specific cell types. To identify the specific receptor mediating lactate-induced macrophage polarization, we analyzed the TNBC single-cell dataset GSE161529 and generated UMAPs (Figure 8A). Among the candidate receptors, GPR65 showed the highest expression in macrophages and was enriched in the macrophage cluster (Figure 8B,C). Consistent with this, lactate treatment markedly upregulated the M2 markers CD163 and CD206 in THP-1–derived macrophages, whereas GPR65 knockdown abolished this induction, reducing CD163 and CD206 to levels comparable to the untreated control (Figure 8D). Taken together, these findings suggest that EIF3H promotes lactate secretion in TNBC, which induces macrophage M2 polarization via the GPR65 receptor.

### 3.9. Tumor Cell-Derived EIF3H Drives Macrophage Polarization via GPR65 to Promote Malignant Progression

**Figure 9:**
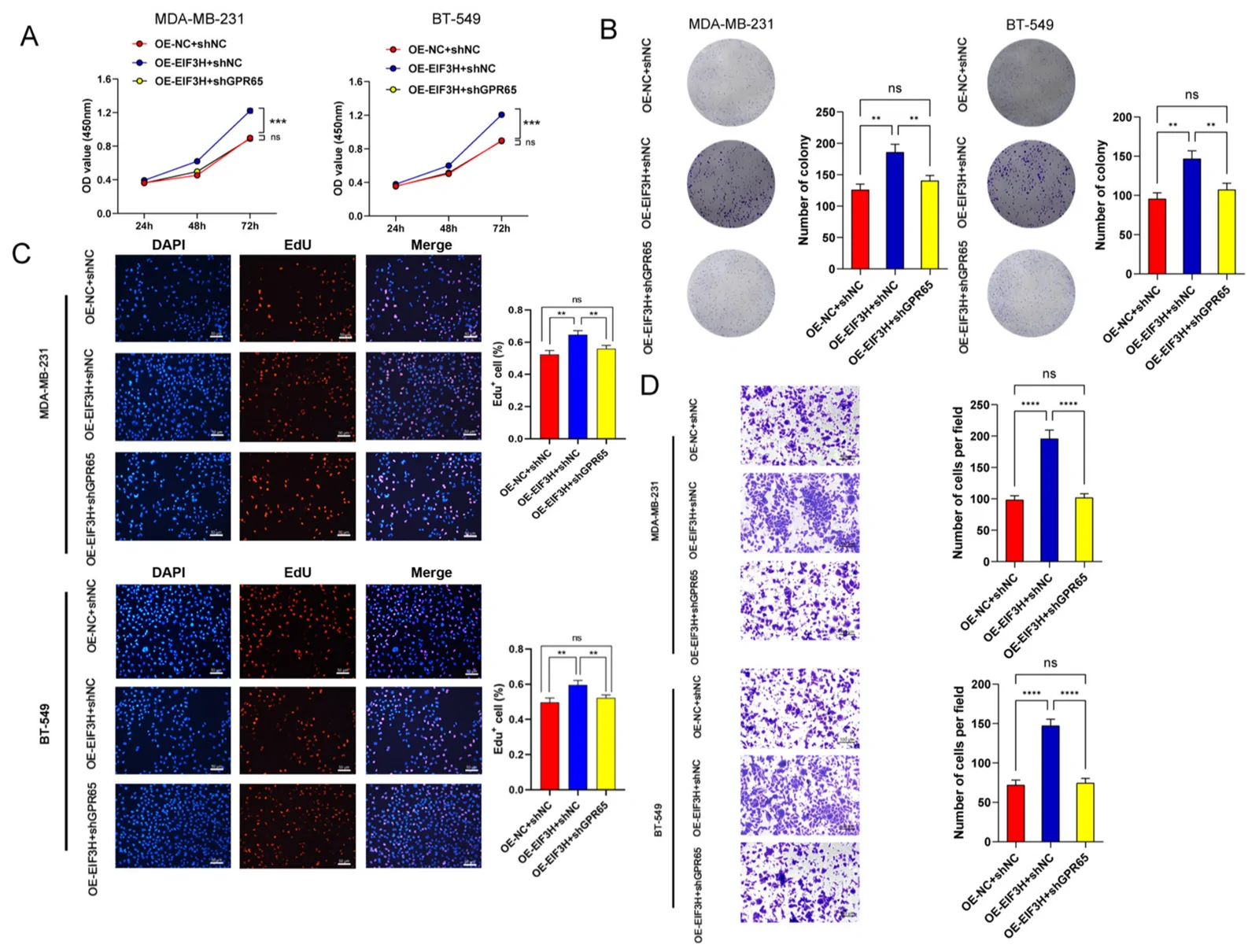
Macrophage GPR65 is required for the pro-tumorigenic feedback of EIF3H-overexpressing TNBC cells. (**A**–**D**) MDA-MB-231 and BT-549 cells were stimulated with conditioned medium (CM) collected from THP-1-derived macrophages with or without GPR65 knockdown; the macrophages had been pre-treated with CM from EIF3H-overexpressing or control (OE-NC) MDA-MB-231 cells. (**A**) Cell proliferation assessed by CCK-8 assay. (**B**) Colony formation assay. (**C**) 5-ethynyl-2′-deoxyuridine (EdU) staining assay. Scale bar, 50 μm. (**D**) Cell migration assessed by Transwell assay. Scale bar = 100 μm. Statistical significance was determined by one-way ANOVA followed by Tukey’s multiple comparisons test; for (**A**), values at the final time point were compared; ** *p* < 0.01, *** *p* < 0.001, **** *p* < 0.0001; ns, not significant.

To further investigate the impact of EIF3H-modulated macrophages on tumor progression, we treated macrophages with conditioned medium (CM) collected from EIF3H-overexpressing or vector control MDA-MB-231 cells, and then collected the macrophage-derived CM to stimulate MDA-MB-231 and BT-549 cells. Macrophages primed with CM from EIF3H-overexpressing cells significantly enhanced the proliferation (CCK-8, colony formation, and EdU assays) and migration (Transwell assay) of both TNBC cell lines compared with controls (Figure 9A–D). To determine whether GPR65 mediates the pro-tumorigenic effects of EIF3H-modulated macrophages, we silenced GPR65 in THP-1-derived macrophages, and the knockdown efficiency was confirmed at both the protein and mRNA levels (Supplementary Figure S5D,F). Importantly, knockdown of GPR65 in macrophages reversed the pro-tumorigenic effects on both proliferation and migration induced by macrophage-derived CM from EIF3H-overexpressing tumor cells to levels comparable to the controls (Figure 9A–D). Together, these findings indicate that tumor cell-derived EIF3H drives M2 macrophage polarization via GPR65, which in turn enhances the malignant behavior of cancer cells.

## 4. Discussion

Extensive genomic and proteomic profiling studies have been conducted in TNBC, and accumulating evidence indicates that aberrant expression of DUBs contributes to the initiation and progression of TNBC [48,49,50]. In addition, the substantial heterogeneity and multiple risk factors characterizing TNBC complicate accurate prognosis prediction, contributing to generally poor clinical outcomes. It therefore remains essential to clarify the pathogenic mechanisms driving TNBC onset and progression [51,52,53]. Protein ubiquitination is an essential post-translational modification that regulates protein degradation and maintains cellular proteostasis. This process is reversible and is continuously governed by the coordinated actions of E3 ubiquitin ligases and deubiquitinating enzymes (DUBs) [54,55]. Growing evidence indicates that DUBs are indispensable to cancer development and progression [56,57,58]. Nonetheless, the specific roles of DUBs in TNBC remain largely undefined. Here, we identify EIF3H as a candidate prognostic biomarker and therapeutic target in TNBC, with elevated EIF3H expression correlating with advanced clinical stage and reduced overall survival (OS) in TNBC patients. Mechanistically, EIF3H promoted tumorigenesis through interactions with LDHA and by regulating its stability, highlighting the therapeutic potential of EIF3H in TNBC. This concept is consistent with complementary strategies that target non-oncogene dependencies in breast cancer: although mechanistically distinct from EIF3H, antisense inhibition of CSA/ERCC8 has been shown to enhance the sensitivity of breast cancer cells, including TNBC, to platinum- and taxane-based chemotherapy [59], supporting the broader notion that targeting adaptive survival pathways is a promising therapeutic strategy.

As a member of the JAMM DUB superfamily, EIF3H governs target-protein stability and function by removing ubiquitin chains across multiple intracellular processes, including endoplasmic reticulum–associated degradation, cell cycle progression, and signal transduction. EIF3H is also recognized as the largest subunit of the eukaryotic translation initiation factor complex [32,33,34]. Numerous studies have demonstrated that EIF3H influences tumor growth, invasion, and metastasis through its involvement in diverse intracellular mechanisms [60,61]. For example, EIF3H drives CRC progression by regulating HAX1 and the RAF1–ERK1/2 signaling pathway [30]. EIF3H interacts with and stabilizes Snail via deubiquitination, thereby promoting Snail-mediated epithelial–mesenchymal transition (EMT) in ESCC [31]. Consistently, TCGA-based analysis revealed a strong association between EIF3H expression and prognosis in TNBC patients, with EIF3H upregulation closely correlated with poor outcomes. Moreover, increased EIF3H expression positively associated with TNBC clinical stage, cell proliferation, and invasiveness. Together, these findings suggest that EIF3H plays a critical role in TNBC tumorigenesis and disease progression.

Metabolic reprogramming via the Warburg effect commonly raises lactate output and drives TME acidosis across cancers, including TNBC [62,63]; lactate itself, once viewed simply as a glycolytic by-product, is now recognized as a signaling molecule with broad roles in tumor progression and disease [64,65,66,67]. Here, we found that EIF3H regulates LDHA ubiquitination and stability in TNBC, such that EIF3H depletion reduced LDHA protein levels and suppressed glycolysis and lactate output. We subsequently explored the mechanism underlying this regulation.

First, EIF3H directly interacts with LDHA. Endogenous EIF3H and LDHA were co-immunoprecipitated from TNBC cells, indicating that this interaction is physiologically relevant. Second, EIF3H regulates LDHA ubiquitination and protein stability. EIF3H knockdown markedly increased LDHA polyubiquitination and reduced LDHA protein levels. After CHX treatment to inhibit protein synthesis, EIF3H depletion shortened the half-life of LDHA, whereas EIF3H overexpression prolonged it. Finally, EIF3H promotes TNBC progression in an LDHA-dependent manner. EIF3H knockdown strongly suppressed TNBC cell proliferation and migration both in vitro and in vivo, and these inhibitory effects were reversed by ectopic LDHA expression. Collectively, these data indicate that EIF3H enhances TNBC proliferation and migration by stabilizing LDHA. Nevertheless, several limitations of our mechanistic analysis should be noted. Although EIF3H contains a JAMM/MPN deubiquitylase domain and our data demonstrate that EIF3H attenuates LDHA polyubiquitination, direct deubiquitination of LDHA by EIF3H was not verified in vitro. Future studies employing deubiquitination assays with purified recombinant proteins, ubiquitin chain-type analysis (e.g., K48- versus K63-linked chains), and mapping of the LDHA lysine residues regulated by EIF3H will be required to fully establish the enzymatic basis of this regulation.

Recent studies have highlighted lactate as a regulator of the immunosuppressive TME, particularly in the functional polarization of tumor-associated macrophages (TAMs), which promote tumor progression through the secretion of cytokines and chemokines [68,69,70]. Previous work reported that tumor-derived lactate induces TAM polarization toward an M2 phenotype [65,71,72]. In our study, lactate metabolism was significantly elevated in TNBC tissues compared with normal tissues. These findings reinforce the view that increased lactate levels play a crucial role in mediating crosstalk between cancer cells and TAM functionality. Proton-sensing G protein-coupled receptors (GPRs), including GPR4, TDAG8 (GPR65), OGR1 (GPR68), and G2A (GPR132), are key mediators of lactate signaling [73,74,75]. Importantly, we found that among these proton-sensing GPRs, GPR65 expression was significantly increased in TNBC tissues compared with normal tissues. Consistent with this observation, scRNA-seq analyses and validation in clinical samples demonstrated that GPR65 was primarily localized to TAMs in TNBC. Subsequent functional experiments further revealed that lactate-induced GPR65 signaling is required for TAM activation. Overall, GPR65 was selectively upregulated on TAMs in TNBC, supporting its potential role in TAM polarization and tumor progression. Regarding the downstream signaling of GPR65, we observed that conditioned medium from control TNBC cells induced STAT6 phosphorylation in THP-1–derived macrophages, whereas this effect was attenuated by conditioned medium from EIF3H-knockdown cells (Figure 7F), implicating STAT6 activation in lactate–GPR65-driven M2 polarization. In glioma, GPR65 has been reported to signal through the cAMP/PKA/CREB cascade in TAMs [71]. Whether additional pathways classically associated with proton-sensing GPCRs, such as ERK and NF-κB, also contribute to GPR65-mediated macrophage polarization in TNBC remains unclear and warrants further investigation. Although the present work focuses on GPR65-mediated signaling, accumulating evidence indicates that lactate may simultaneously regulate tumor progression through protein lactylation [76,77], histone lactylation [78] and metabolic signaling [72]. Therefore, these mechanisms should be acknowledged as potentially complementary rather than mutually exclusive.

In summary, our study comprehensively evaluated the role of EIF3H in TNBC progression and metastasis using patient tissue analyses as well as in vitro and in vivo experiments. Elevated EIF3H expression was identified as a characteristic feature of TNBC and correlated with poor prognosis. EIF3H promoted TNBC progression by interacting with LDHA, reducing its ubiquitination and enhancing its stability. Stabilized LDHA enhanced lactate production, which in turn promoted TAM polarization via GPR65. Genetic interference with EIF3H significantly suppressed tumor progression. To the best of our knowledge, this is the first study to define a metabolic regulatory function of EIF3H in TNBC. Importantly, our findings provide evidence-based insights to support the development of EIF3H-targeted diagnostic and therapeutic strategies for TNBC.

## 5. Conclusions

This study identifies EIF3H as a critical driver of TNBC progression, linking the regulation of protein ubiquitination to metabolic reprogramming and immune modulation. Transcriptionally activated by TRPS1, EIF3H stabilizes LDHA by attenuating its ubiquitination, enhancing glycolysis and lactate production to sustain tumor cell proliferation and migration. Tumor-derived lactate, in turn, polarizes tumor-associated macrophages toward the M2 phenotype via GPR65, forming a feedback loop that sustains malignant progression. Genetic interference with EIF3H markedly suppressed tumor growth both in vitro and in vivo.

Together, these findings define a previously unrecognized metabolic role for EIF3H and establish the TRPS1–EIF3H–LDHA axis, together with lactate–GPR65 signaling, as a mechanistic framework underlying TNBC progression. Beyond its value as a prognostic biomarker, EIF3H represents a promising therapeutic target. Future work evaluating pharmacological inhibition of EIF3H, alone or combined with existing therapies, will be needed to translate these findings into clinical benefit for patients with TNBC.

## Data Availability

The data presented in this study are available on request from the corresponding author. The data are not publicly available due to ethical restrictions and patient privacy protection requirements.

## Supplementary Materials

The following supporting information can be downloaded at: https://www.mdpi.com/article/10.3390/cancers18172735/s1, Figure S1: TRPS1 regulates EIF3H transcription; Figure S2: EIF3H overexpression promotes tumor progression; Figure S3: Restoration of LDHA rescues the impaired proliferation and migration of EIF3H-knockdown TNBC cells; Figure S4: LDHA/SLC16A1 expression, prognosis, and EIF3H glycolytic function in breast cancer; Figure S5: Validation of lentiviral-mediated EIF3H overexpression, EIF3H knockdown, LDHA knockdown in TNBC cells and GPR65 knockdown efficiency in THP-1-derived macrophages; Figure S6: Original Western blot images; Table S1: Clinical characteristics of the patient cohort; Table S2: List of genes correlated with EIF3H expression (UALCAN); Table S3: List of genes correlated with EIF3H expression (METABRIC); Table S4: Intersection of EIF3H-correlated genes between UALCAN-TNBC and METABRIC cohorts.

## Abbreviations

2-DG: 2-deoxy-D-glucose; ANOVA: analysis of variance; BCA: bicinchoninic acid; BSA: bovine serum albumin; CC: confidence interval; CNA: copy number alteration; CCK-8: cell counting kit-8; ChIP: chromatin immunoprecipitation; CM: conditioned medium; Co-IP: co-immunoprecipitation; CPTAC: Clinical Proteomic Tumor Analysis Consortium; DAPI: 4′,6-diamidino-2-phenylindole; DMEM: Dulbecco’s modified Eagle medium; DMFS: distant metastasis-free survival; DUBs: deubiquitinating enzymes; ECAR: extracellular acidification rate; EdU: 5-ethynyl-2′-deoxyuridine; ECL: enhanced chemiluminescence; EMT: epithelial–mesenchymal transition; ER: estrogen receptor; FBS: fetal bovine serum; GPRs: G-protein coupled receptors; HA: hemagglutinin; HR: hazard ratio; IACUC: Institutional Animal Care and Use Committee; ICQ: intensity correlation quotient; IHC: immunohistochemistry; IgG: immunoglobulin G; IP/MS: immunoprecipitation coupled with mass spectrometry; LDH: lactate dehydrogenase; LDHA: lactate dehydrogenase A; MCT1: monocarboxylate transporter 1; OD: optical density; OS: overall survival; PCC: Pearson correlation coefficient; PFA: paraformaldehyde; PKM2: pyruvate kinase M2; PMA: phorbol 12-myristate 13-acetate; PMSF: phenylmethylsulfonyl fluoride; PR: progesterone receptor; PVDF: polyvinylidene difluoride; qRT-PCR: quantitative real-time PCR; RPMI: Roswell Park Memorial Institute; SD: standard deviation; SDS-PAGE: sodium dodecyl sulfate–polyacrylamide gel electrophoresis; SEM: standard error of the mean; shEIF3H group: stably knocked down EIF3H; shNC group: stably transfecting the empty vector; SRCC: Spearman rank correlation coefficient; TAMs: tumor-associated macrophages; TBST: tris-buffered saline with Tween 20; TCGA: The Cancer Genome Atlas; TME: tumor microenvironment; TNBC: triple-negative breast cancer; UMAP: uniform manifold approximation and projection; UPS: ubiquitin–proteasome system; WB: Western blot.
