# Supplementary material for: EIF3H Modulates Glycolysis Through LDHA Stabilization in Triple-Negative Breast Cancer
Source: Cancers (Basel). 2026 Aug 23;18(17):2735. doi: 10.3390/cancers18172735 (PMC13564525; doi:10.3390/cancers18172735)
Supplement: Supplementary file 1 [file cancers-18-02735-s001.zip › cancers-4474273-supplementary.pdf]

Supplementary Materials for

EIF3H Modulates Glycolysis through LDHA Destabilization in Triple-Negative Breast Cancer

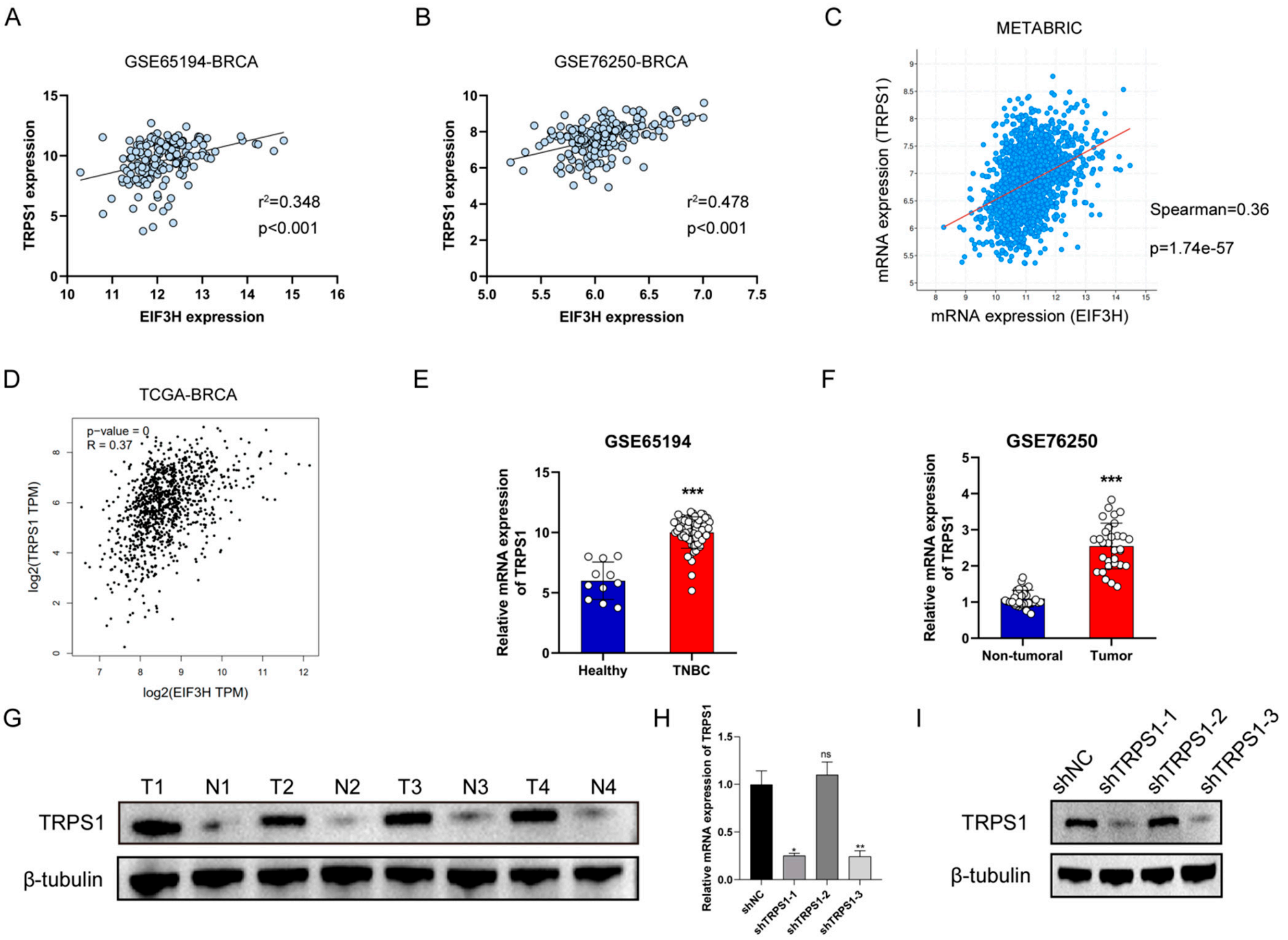

Supplementary Figure S1 TRPS1 regulates EIF3H transcription.

(A) TRPS1 expression was correlated with EIF3H expression in the GSE65194-BRCA cohort. (B) TRPS1 expression was correlated with EIF3H expression in the GSE76250-BRCA cohort. (C) TRPS1 mRNA expression was correlated with EIF3H mRNA expression in the METABRIC cohort. (D) TRPS1 mRNA expression was correlated with EIF3H mRNA expression in the TCGA-BRCA cohort. (E) TRPS1 mRNA expression levels in GSE65194 database. (F) TRPS1 mRNA expression levels in GSE76250 database. (G) TRPS1 protein expression levels in tumor and adjacent tissue of TNBC. (H) Knockdown efficiency of three independent shRNAs targeting TRPS1 in MDA-MB-231 cells, assessed by qRT-PCR. (I) Knockdown efficiency of three independent shRNAs targeting TRPS1 in MDA-MB-231 cells, assessed by western blotting. The original uncropped Western blot

images for panel G and I are shown in Supplementary Figure S6 (Original Images 28–29 and 38-39，respectively).Statistical significance was determined by unpaired two-tailed Student's t-test, *$p < 0.05$, **$p < 0.01$, ***$p < 0.001$.

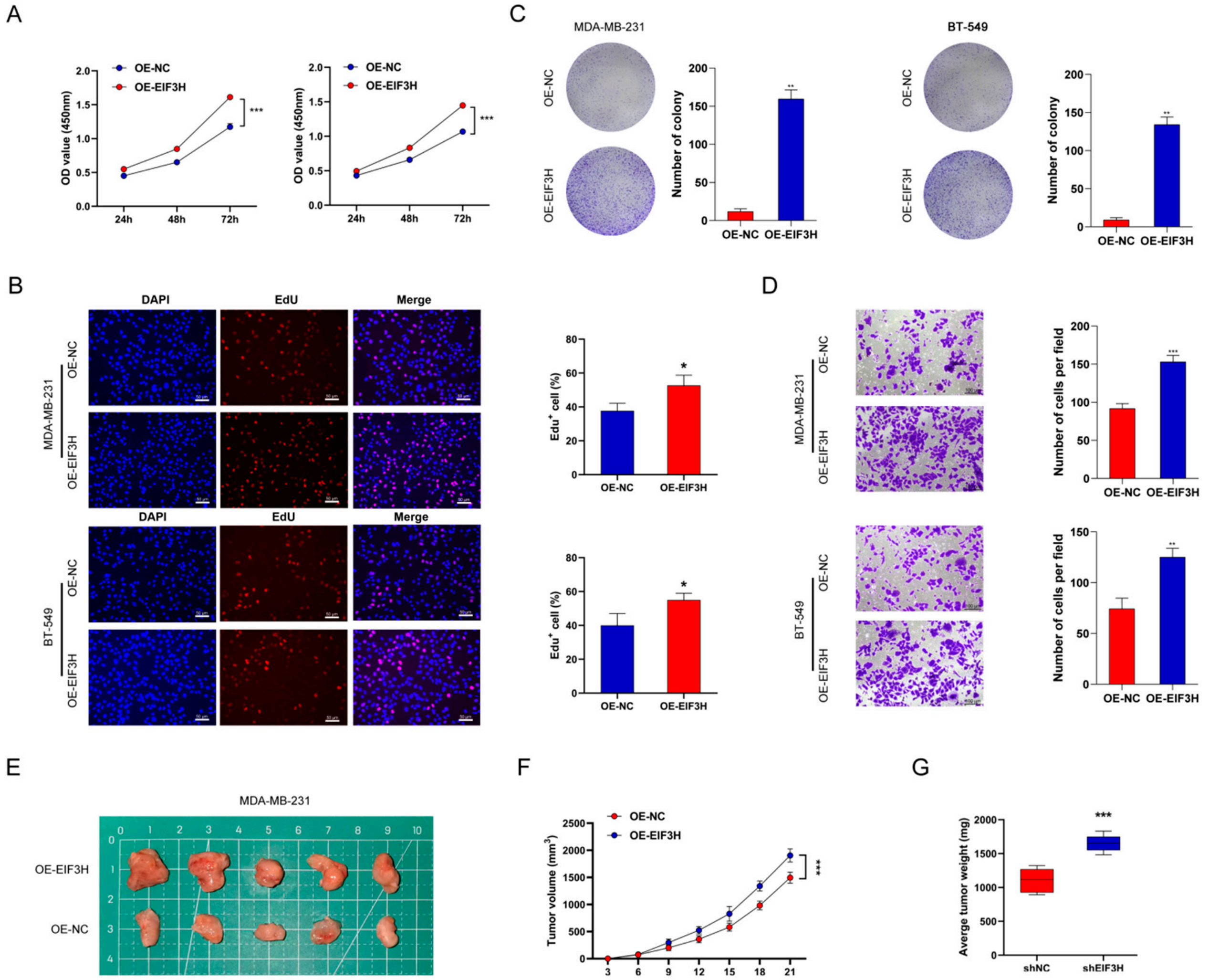

Supplementary Figure S2 EIF3H-Overexpression promotes tumor progression

(A) Proliferation of MDA-MB-231 and BT-549 cells with overexpression of EIF3H (0E-EIF3H) or negative control (OE-NC). (B). 5-ethynyl-2'-deoxyuridine (EdU) staining assays of MDA-MB-231 and BT-549 cells with overexpression of EIF3H (0E-EIF3H) or negative control (OE-NC). (C) Colony formation assay of MDA-MB-231 and BT-549 cells with overexpression of EIF3H (0E-EIF3H) or negative control (OE-NC). (D) Migration of MDA-MB-231 and BT-549 cells with overexpression of EIF3H (OE-EIF3H) or negative control (OE-NC), assessed by transwell assay. Scale bar = 50 µm. (E). Representative subcutaneous tumors collected

from BALB/c nude mice. (F) Tumor proliferation curves of subcutaneous xenografts in MDA-MB-231-bearing BALB/c nude mice. (G). TRPS1 protein expression levels in MDA-MB-231 cell line. (H) Tumors weight of subcutaneous xenografts in MDA-MB-231-bearing BALB/c nude mice. Statistical significance was determined by unpaired two-tailed Student's t-test; for (A, F), values at the final time point were compared; $*p < 0.05$, $**p < 0.01$, $***p < 0.001$.

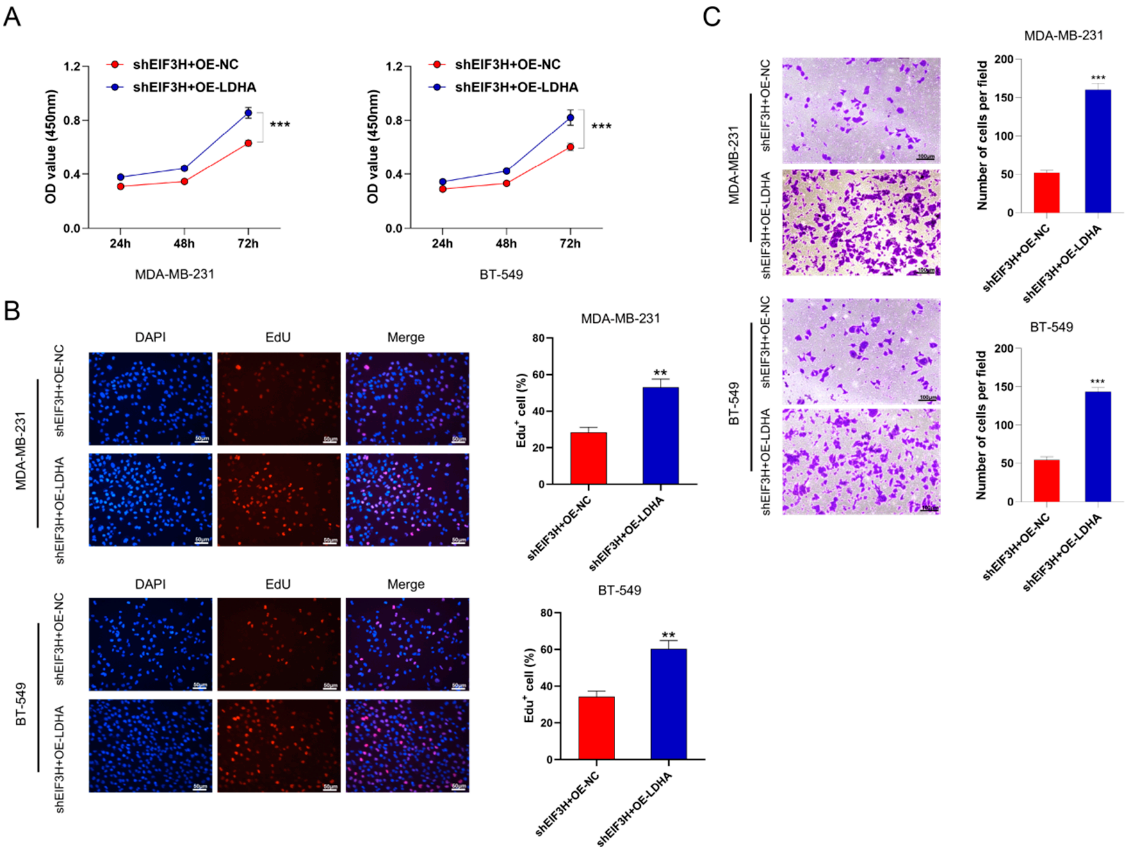

Supplementary Figure S3. Restoration of LDHA rescues the impaired proliferation and migration of EIF3H-knockdown TNBC cells

(A–C) EIF3H-knockdown MDA-MB-231 and BT-549 cells were transduced with LDHA-overexpression lentivirus (OE-LDHA) or negative control (OE-NC). (A) Cell proliferation assessed by CCK-8 assay. (B) 5-Ethynyl-2'-deoxyuridine (EdU) staining assay. Scale bar = 50 μm. (C) Transwell migration assay. Scale bar = 100 μm. Statistical significance was determined by unpaired two-tailed Student's t-test; $*p < 0.05$, $**p < 0.01$, $***p < 0.001$.

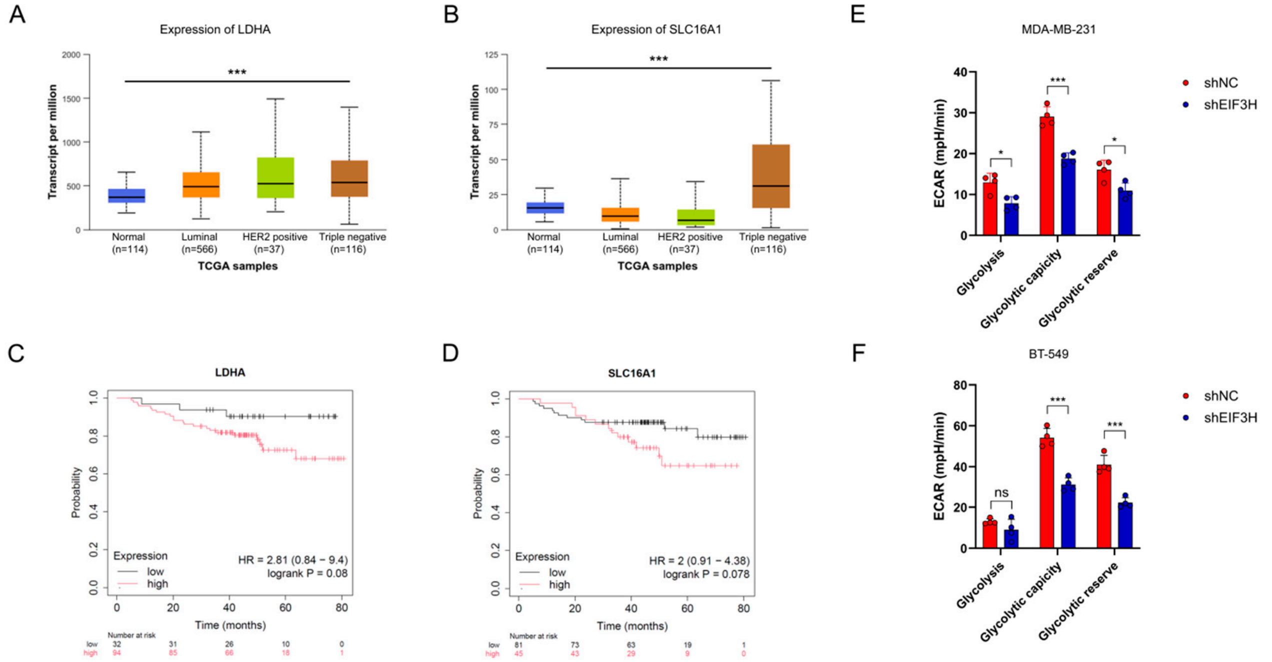

Supplementary Figure S4 LDHA/SLC16A1 expression, prognosis, and EIF3H glycolytic function in breast cancer

(A) The mRNA levels of LDHA were analyzed using bulk RNA-seq data from the TCGA. (B) The mRNA levels of MCT1 (referred as SLC16A1 at the mRNA level) were analyzed using bulk RNA-seq data from the TCGA. (C) Kaplan–Meier analysis results revealed that high LDHA expression correlated with poor overall survival (OS) among TNBC patients (D) Kaplan–Meier analysis results revealed that high SLC16A1 expression correlated with poor overall survival (OS) among TNBC patients. (E, F) Quantification of glycolysis, glycolytic capacity, and glycolytic reserve from the Seahorse XF Glycolysis Stress Test ECAR profiles (Figure 7D) in MDA-MB-231 (E) and BT-549 (F) cells. Data are presented as mean ± *SD* of $n = 4$ wells per group; two-tailed unpaired Student's t-test; ns, not significant, $*p < 0.05$, $**p < 0.01$, $***p < 0.001$.

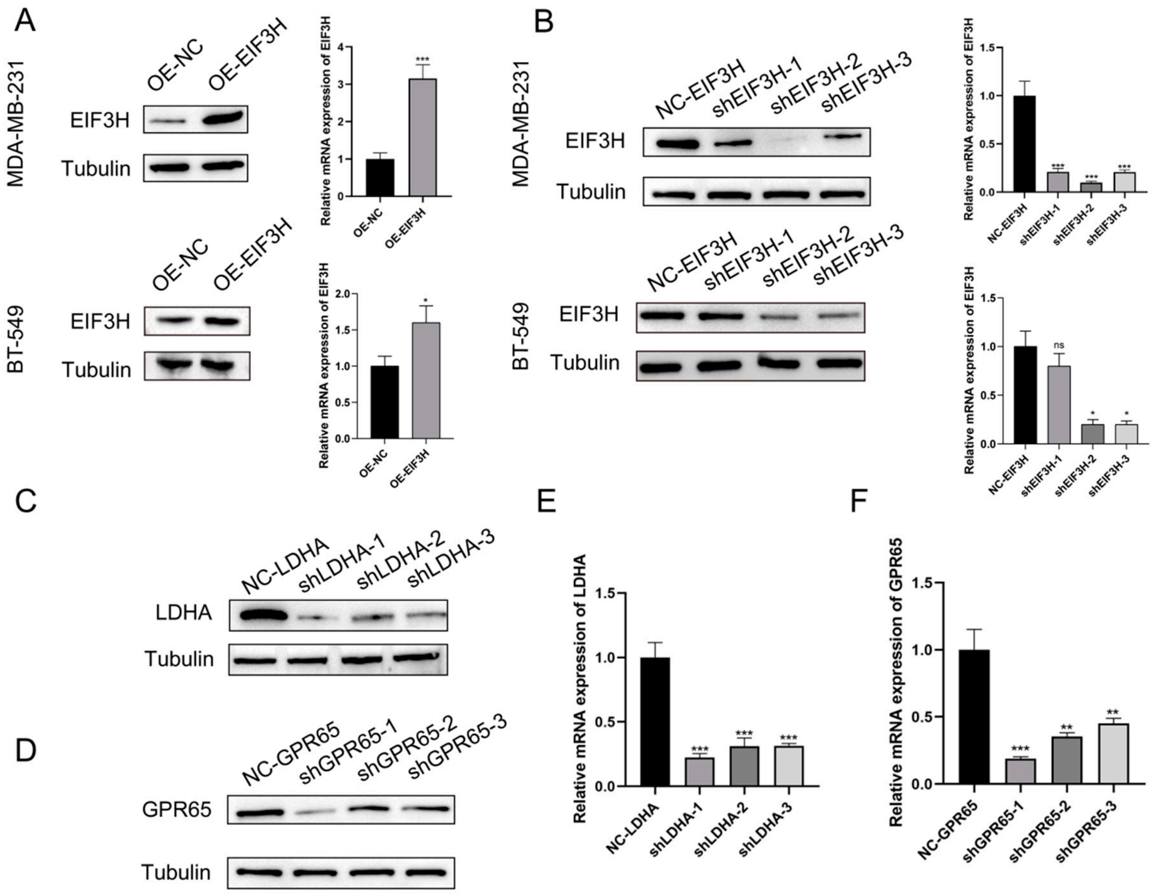

Supplementary Figure S5 Validation of lentiviral-mediated EIF3H overexpression, EIF3H knockdown, LDHA knockdown in TNBC cells and GPR65 knockdown efficiency in THP-1-derived macrophages

(A) Western blot and qRT-PCR analysis of EIF3H overexpression (OE-EIF3H vs. OE-NC) in MDA-MB-231 and BT-549 cells. (B) Western blot and qRT-PCR analysis of the knockdown efficiency of three independent shRNAs targeting EIF3H (shEIF3H-1, shEIF3H-2, and shEIF3H-3) versus negative control (shNC) in MDA-MB-231 and BT-549 cells. shEIF3H-2 exhibited the highest knockdown efficiency in both cell lines and was used for all subsequent functional experiments. (C) Western blot analysis of LDHA protein expression in MDA-MB-231 cells transfected with three independent LDHA shRNAs (shLDHA-1, shLDHA-2, shLDHA-3) or negative control (NC-LDHA). (D) Western blot analysis of GPR65 protein expression in THP-1-derived macrophages transfected with three independent GPR65 shRNAs (shGPR65-1, shGPR65-2, shGPR65-3) or negative control (NC-GPR65). (E) Quantitative RT-PCR analysis of LDHA mRNA expression in MDA-MB-231 cells transfected with shLDHA-1/2/3 or NC-LDHA. (F) Quantitative RT-PCR analysis of GPR65 mRNA expression in THP-1-derived macrophages transfected with shGPR65-1/2/3 or NC-GPR65. β-Tubulin was used as the loading control for all Western blot experiments. Data are presented as mean ± *SD*. Statistical

significance was determined by unpaired two-tailed Student's t-test (A) or one-way ANOVA followed by Dunnett's multiple comparisons test (B, E, F); *$p < 0.05$, **$p < 0.01$, ***$p < 0.001$, compared with the corresponding negative control group. The original uncropped Western blot images for panels A–D are shown in Supplementary Figure S6 (Original Images 30–37).

Supplementary Figure S6 Original WB images

Original Image 1. Figure 2G anti-EIF3H

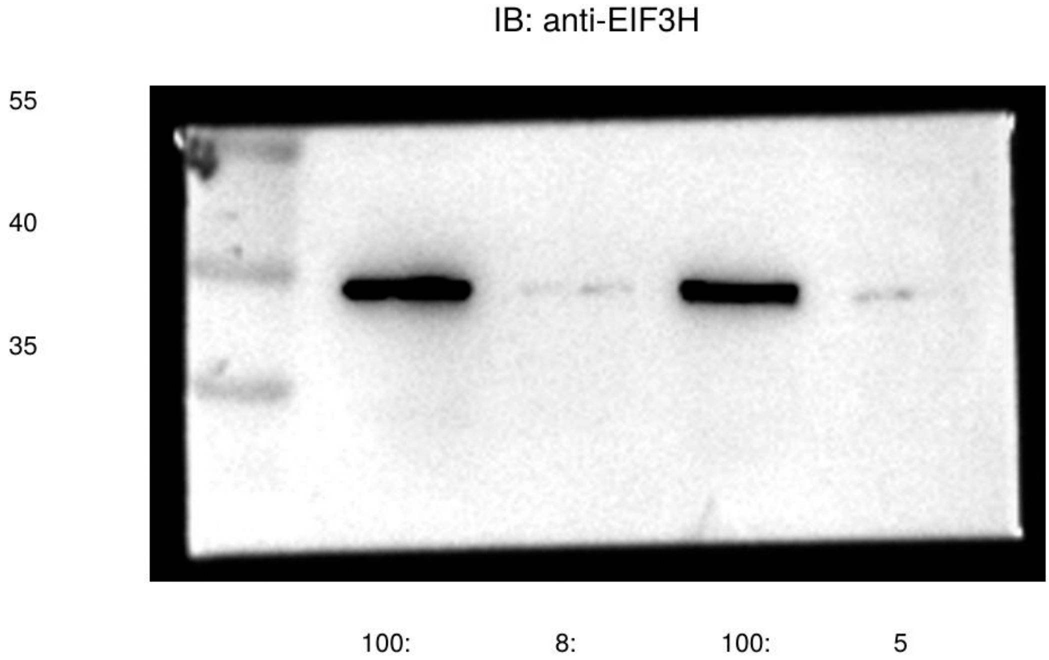

Original Image 2. Figure 2G anti- -Tubulin

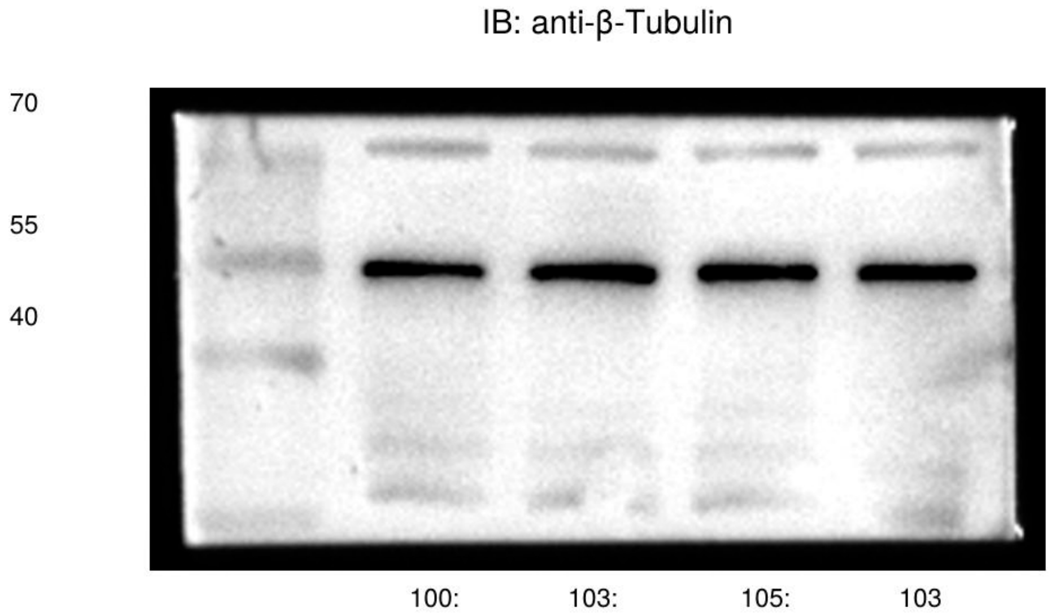

Original Image 3. Figure 4C   anti-EIF3H (Co-IP)

IB: anti-EIF3H (Co-IP)

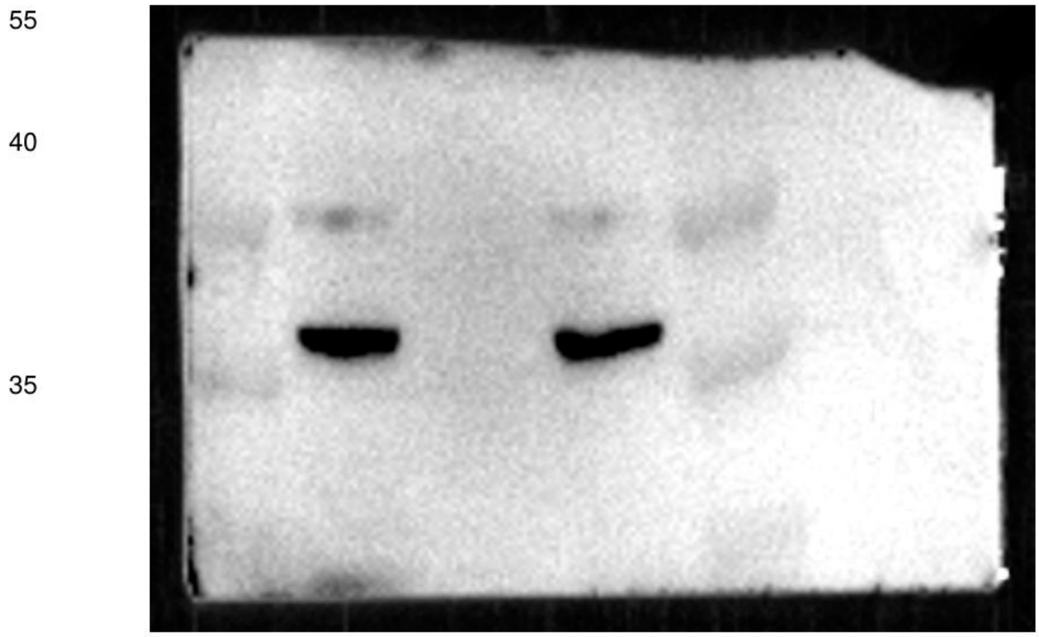

Original Image 4. Figure 4C   anti-LDHA (Co-IP)

IB: anti-LDHA (Co-IP)

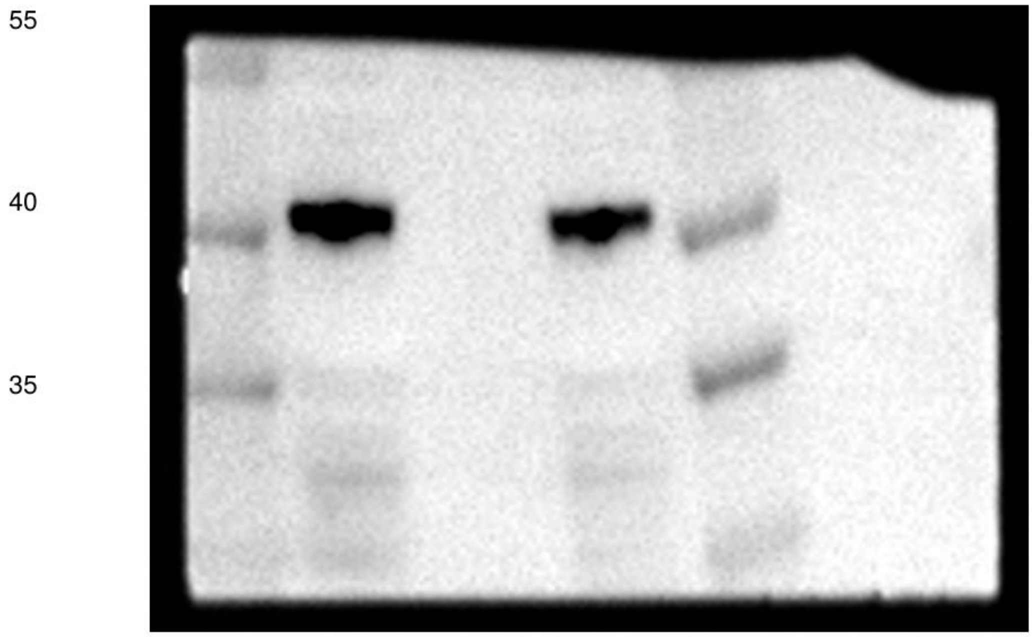

Original Image 5. Figure 5A   anti-EIF3H (OE-NC vs OE-EIF3H)

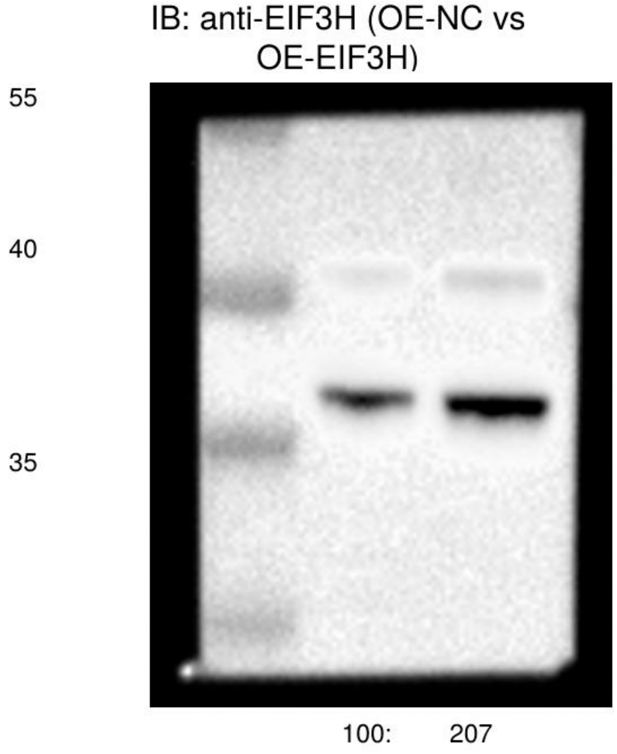

Original Image 6. Figure 5A anti- -Tubulin (OE-NC vs OE-EIF3H)

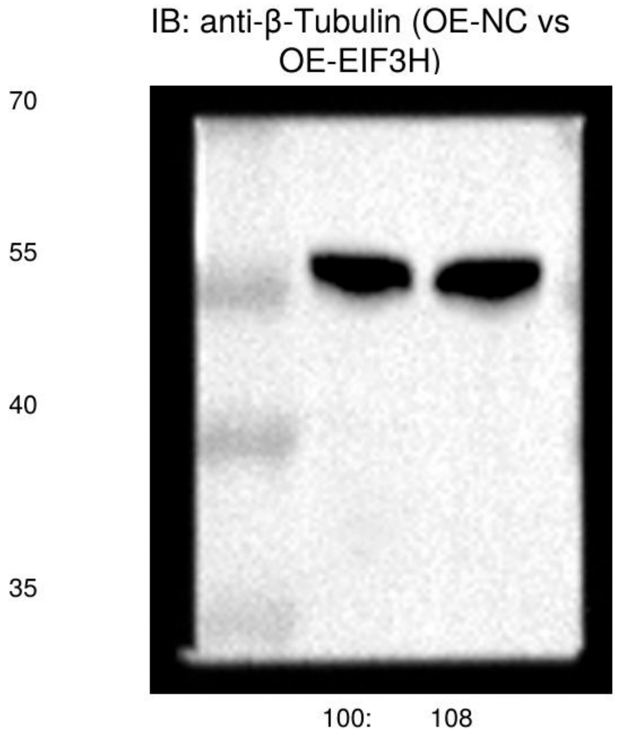

Original Image 7. Figure 5A anti-EIF3H (shNC vs shEIF3H)

IB: anti-EIF3H (shNC vs shEIF3H)

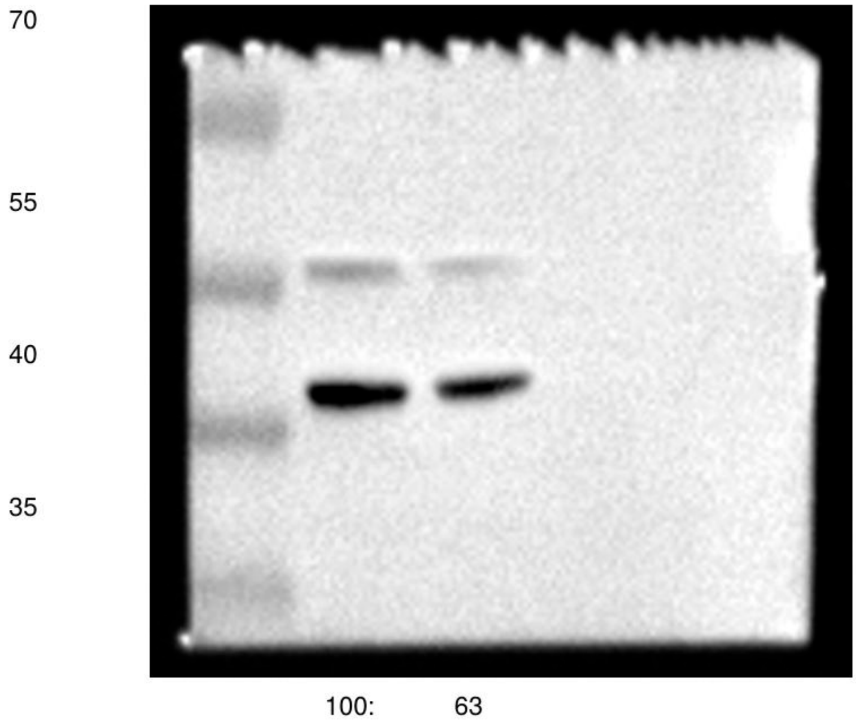

Original Image 8. Figure 5A   anti- -Tubulin (shNC vs shEIF3H)

IB: anti-β-Tubulin (shNC vs shEIF3H)

70

55

40

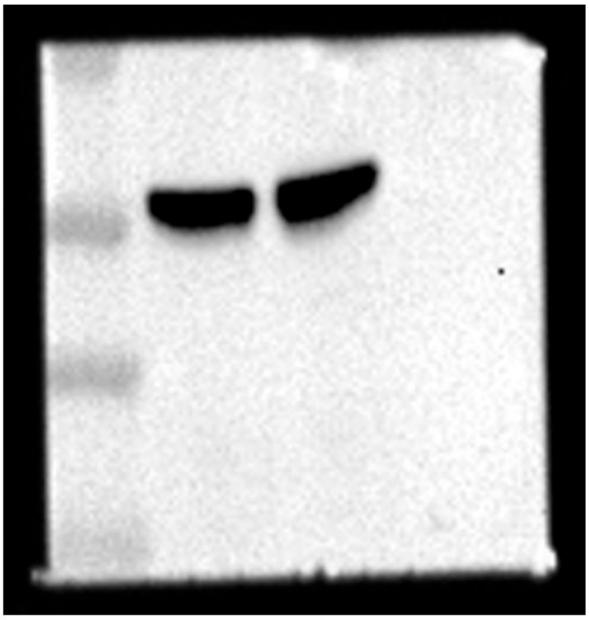

100:        105

Original Image 9. Figure 5B anti-EIF3H (CHX chase, OE-NC/OE-EIF3H)

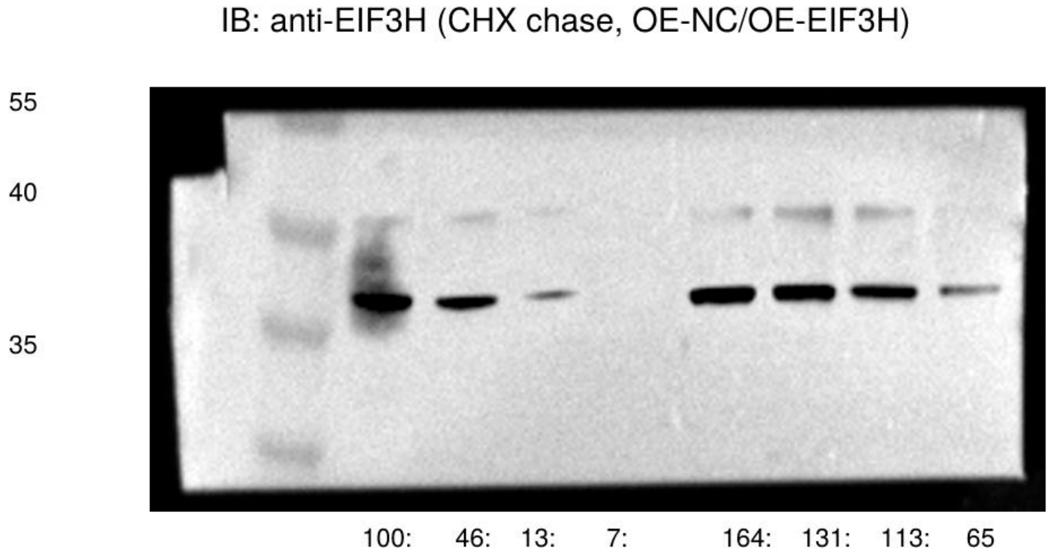

Original Image 10. Figure 5B anti-LDHA (CHX chase, OE-NC/OE-EIF3H)

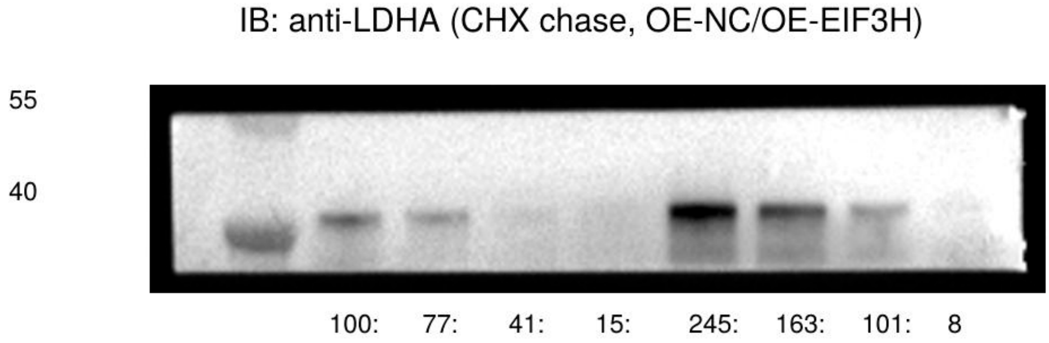

Original Image 11. Figure 5B anti- -Tubulin (CHX chase, OE-NC/OE-EIF3H)

IB: anti-β-Tubulin (CHX chase, OE-NC/OE-EIF3H)

55

40

35

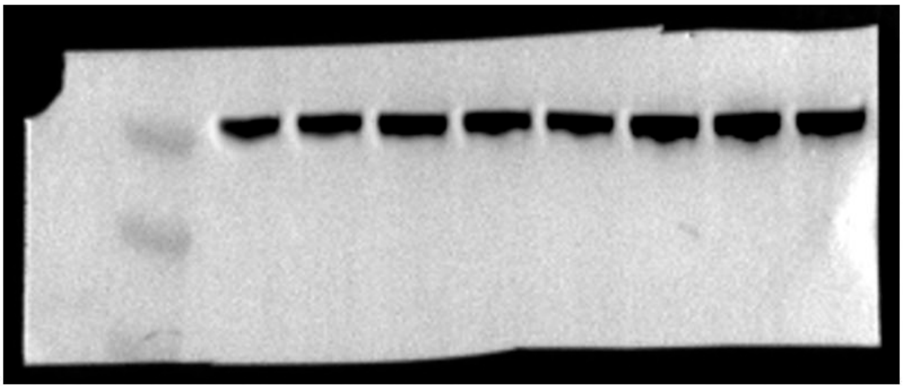

100: 101: 102: 99: 103: 97: 98: 104

Original Image 12. Figure 5C anti-EIF3H (CHX chase, shNC/shEIF3H)

IB: anti-EIF3H (CHX chase, shNC/shEIF3H)

55

40

35

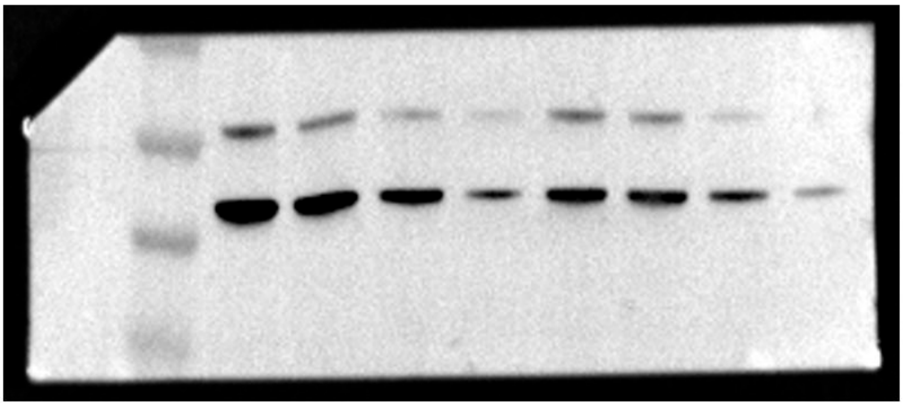

100: 77: 52: 19: 42: 35: 18: 11

Original Image 13. Figure 5C anti-LDHA (CHX chase, shNC/shEIF3H)

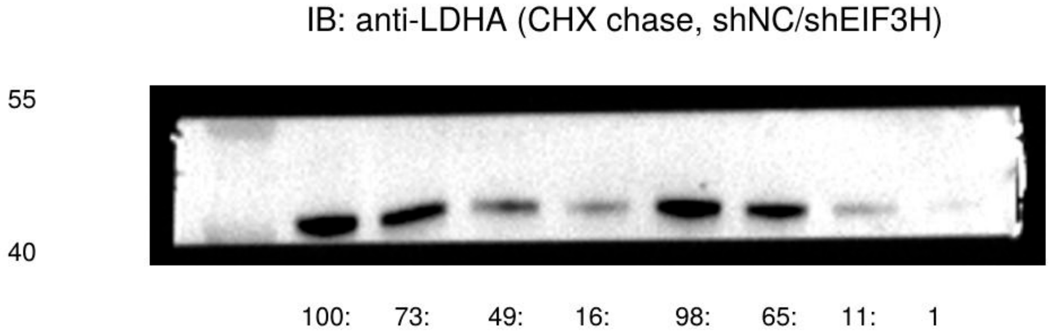

Original Image 14. Figure 5C anti- -Tubulin (CHX chase, shNC/shEIF3H)

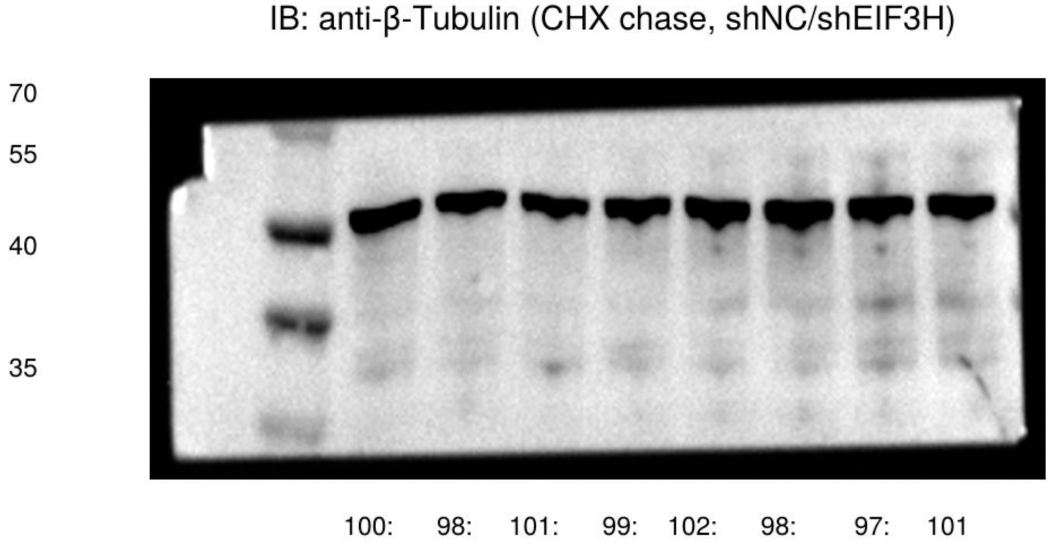

Original Image 15. Figure 5D   anti-EIF3H

IB: anti-EIF3H

40

35

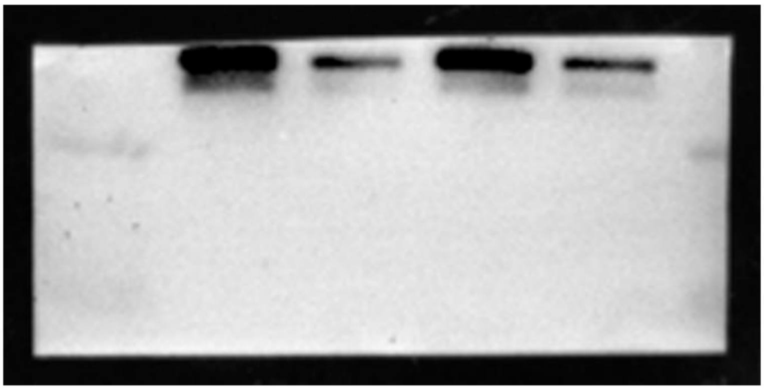

100: 14: 97: 19

Original Image 16. Figure 5D   anti-LDHA

IB: anti-LDHA

55

40

35

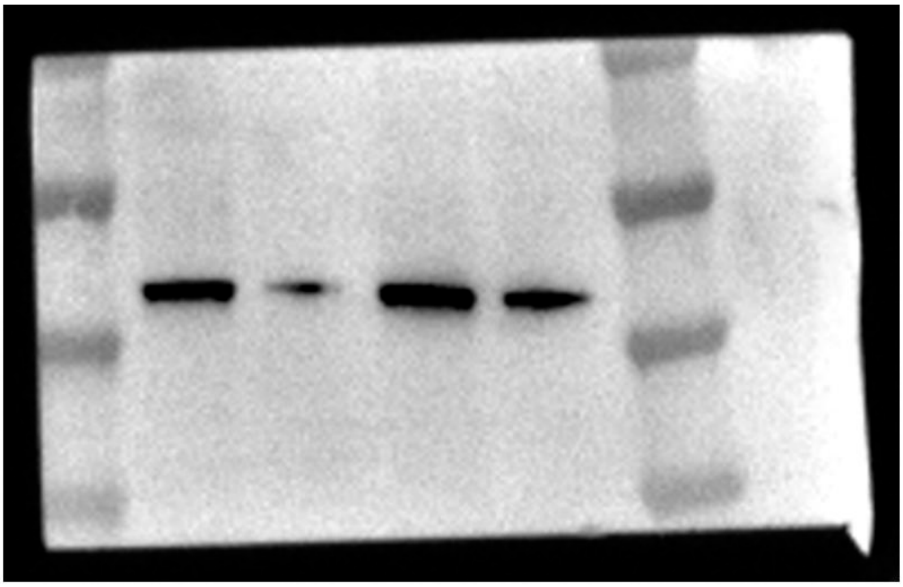

100: 6: 161: 58

Original Image 17. Figure 5D anti- -Tubulin

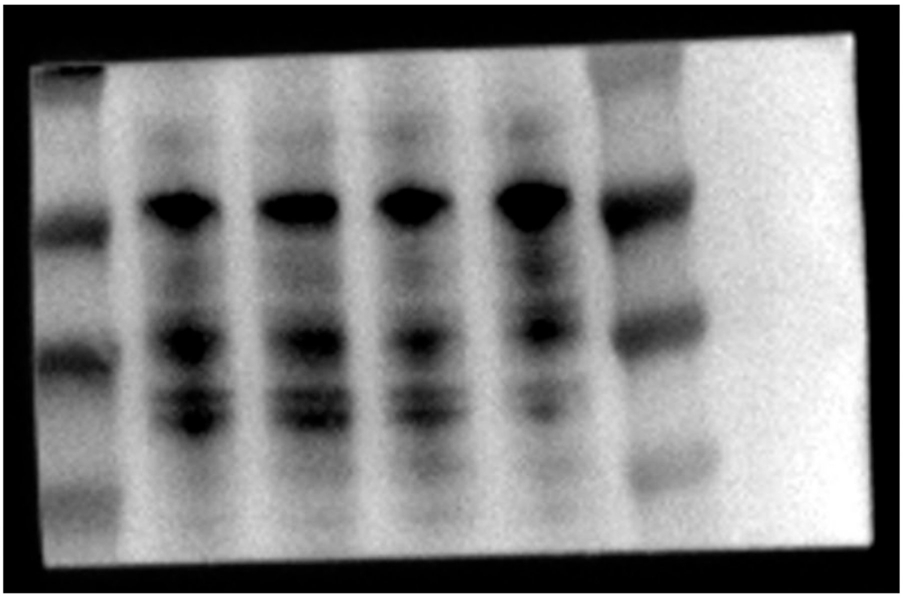

Original Image 18. Figure 5E anti-EIF3H (input)

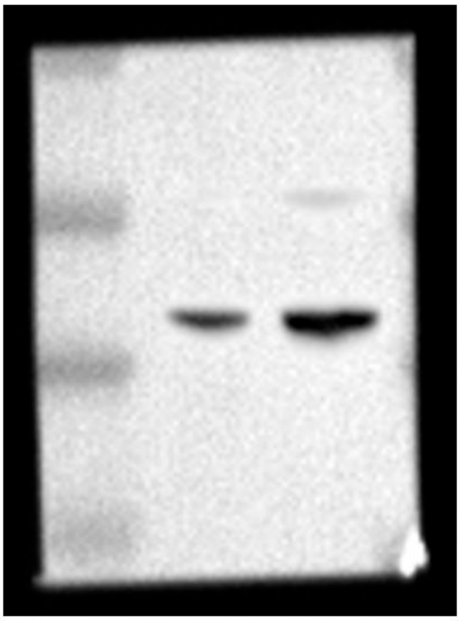

Original Image 19. Figure 5E   anti-LDHA (IP)

IB: anti-LDHA (IP)

55
40
35

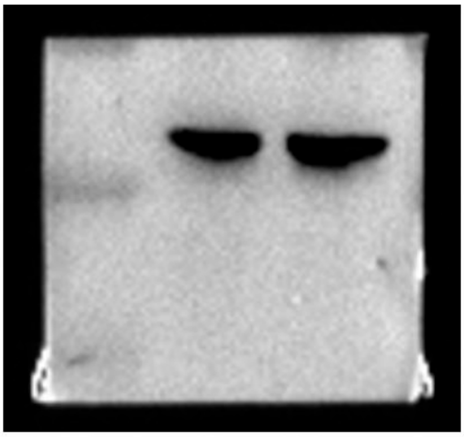

100: 94

Original Image 20. Figure 5E   anti-LDHA (input)

IB: anti-LDHA (input)

55
40
35

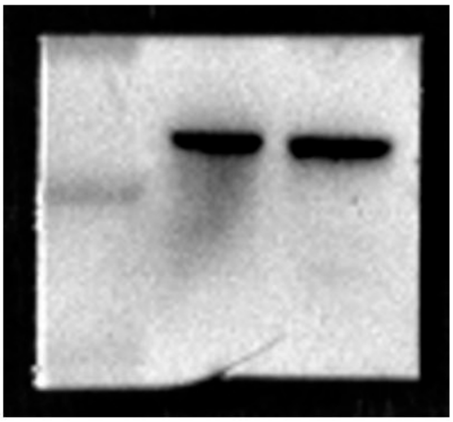

100: 97

Original Image 21. Figure 5E anti- -Tubulin (input)

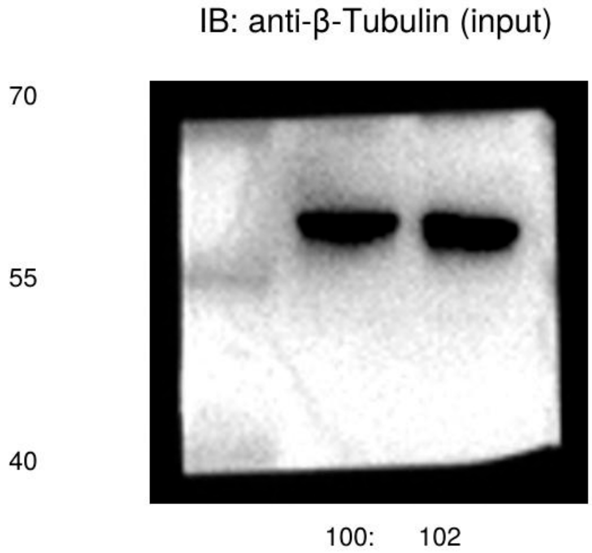

Original Image 22. Figure 5E anti-Ubiquitin (Ub-LDHA)

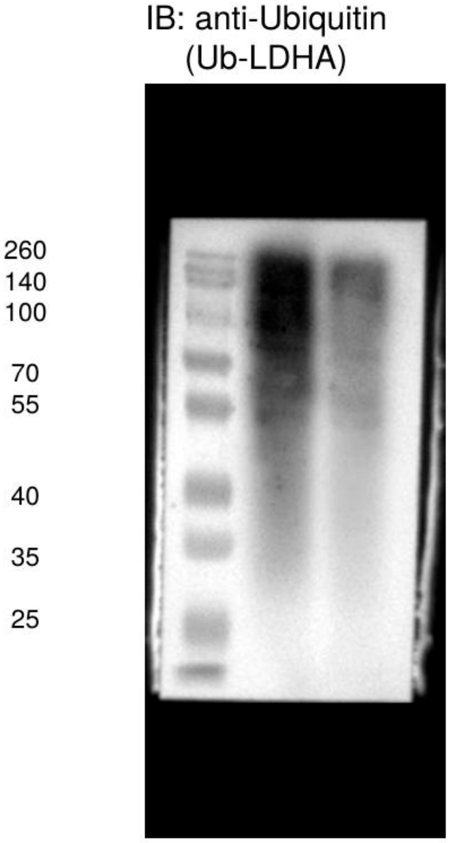

Original Image 23. Figure 5F anti-EIF3H (input)

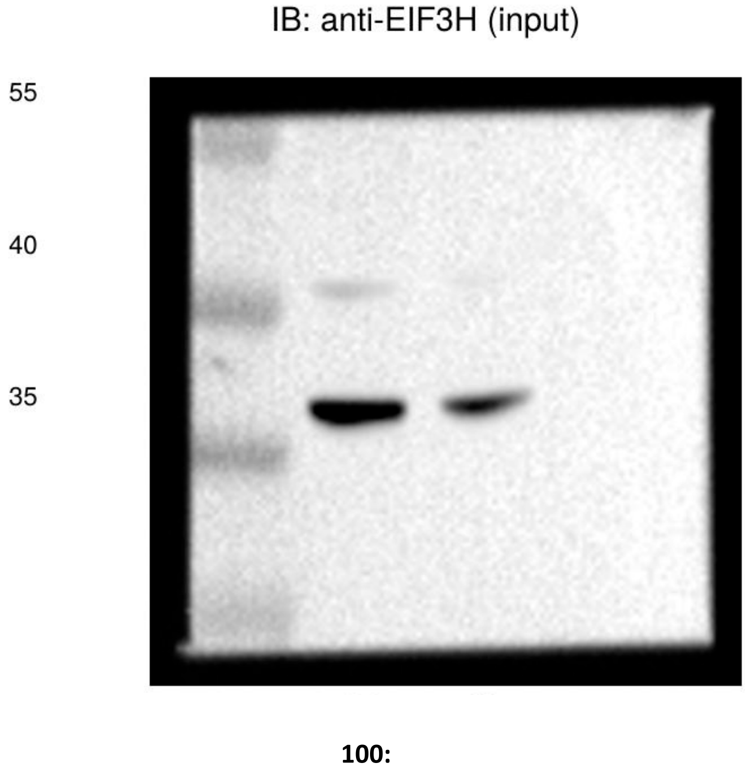

Original Image 24. Figure 5F anti-LDHA (IP)

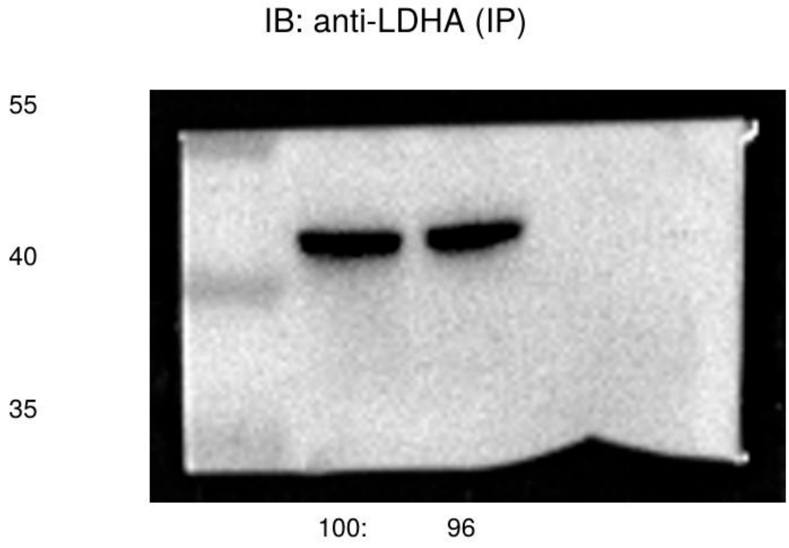

Original Image 25. Figure 5F anti-LDHA (input)

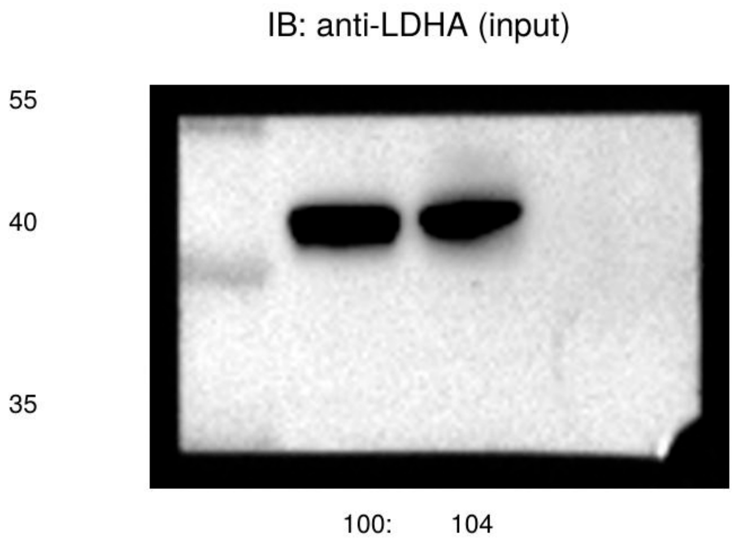

Original Image 26. Figure 5F anti- -Tubulin (input)

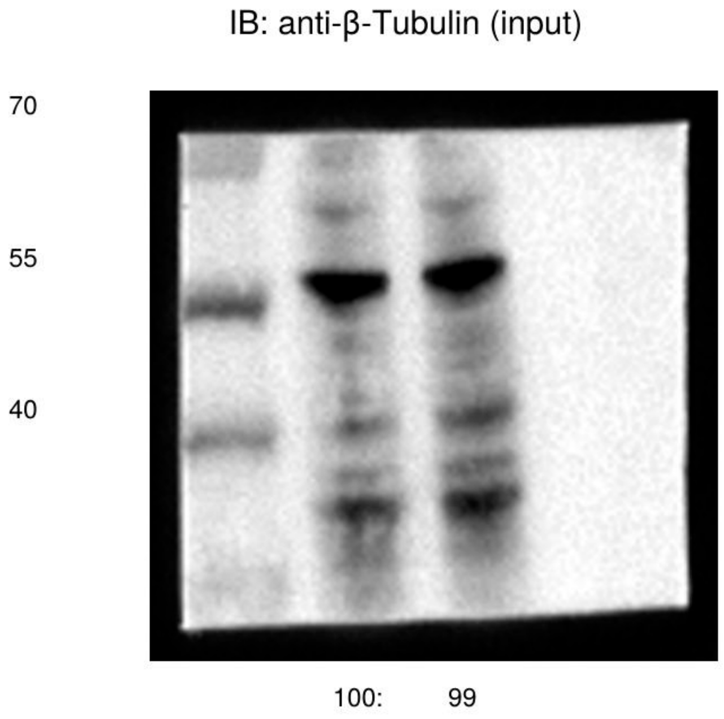

Original Image 27. Figure 5F   anti-Ubiquitin (Ub-LDHA)

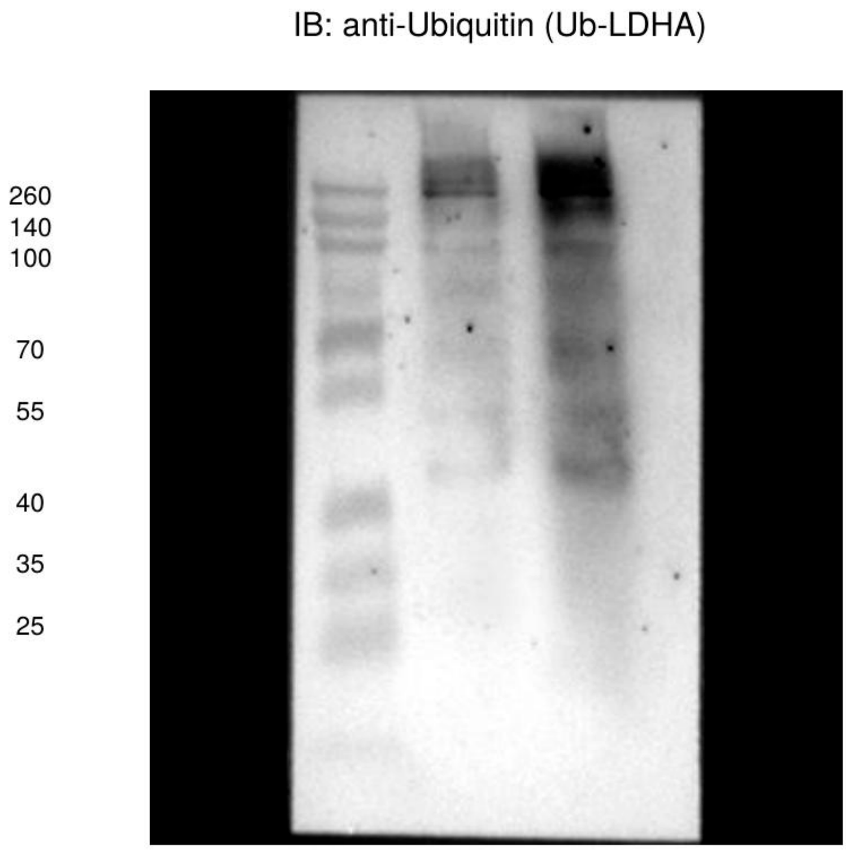

Original Image 28. Supplementary Figure S1G   anti-TRPS1

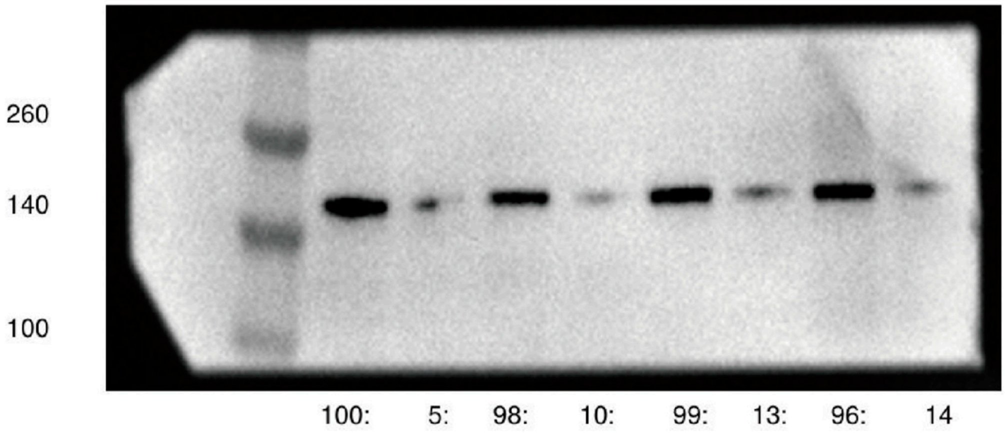

Original Image 29. Supplementary Figure S1G   anti- -Tubulin

IB: anti-β-Tubulin

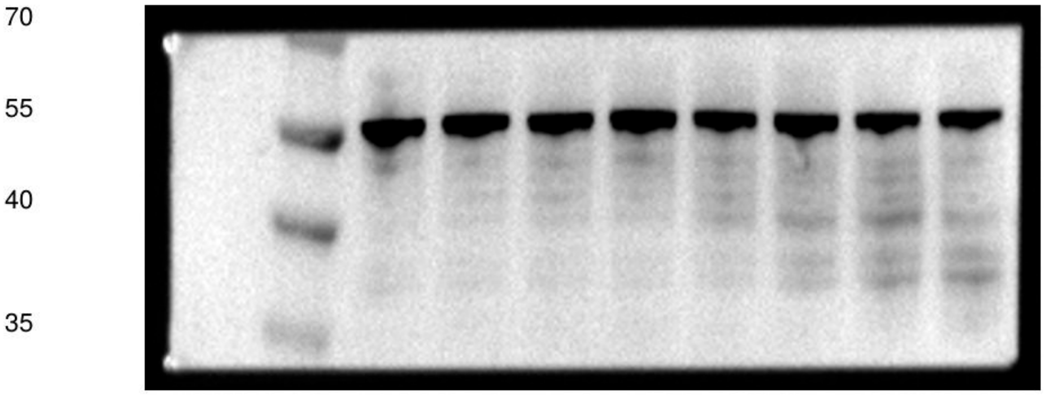

100:    97:   102:   105:   99:   100:   103:   96

Original Image 30. Supplementary Figure S4A   anti-EIF3H (OE-NC vs OE-EIF3H)

IB: anti-EIF3H (OE-NC vs OE-EIF3H)

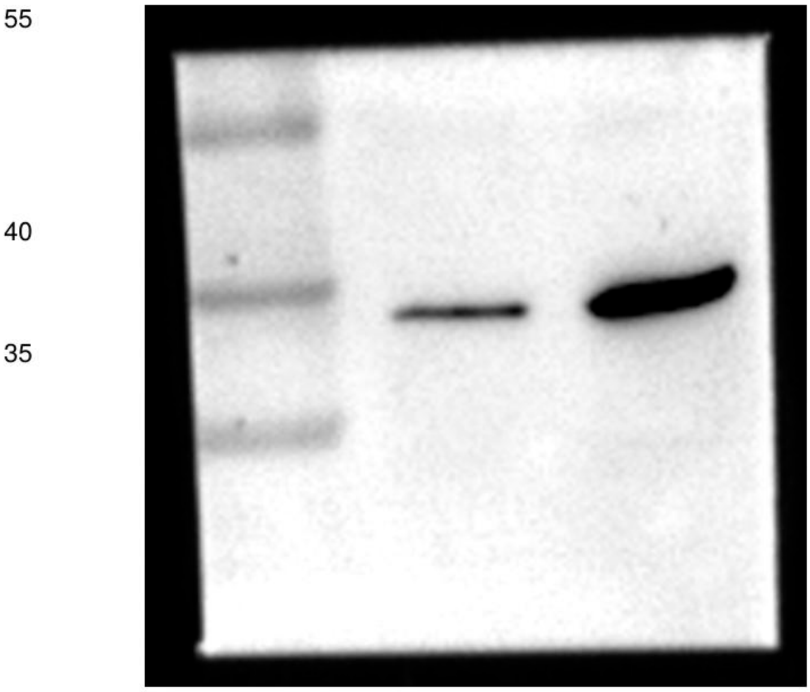

100:    418

Original Image 31. Supplementary Figure S4A anti- -Tubulin (OE-NC vs OE-EIF3H)

IB: anti-β-Tubulin (OE-NC vs OE-EIF3H)

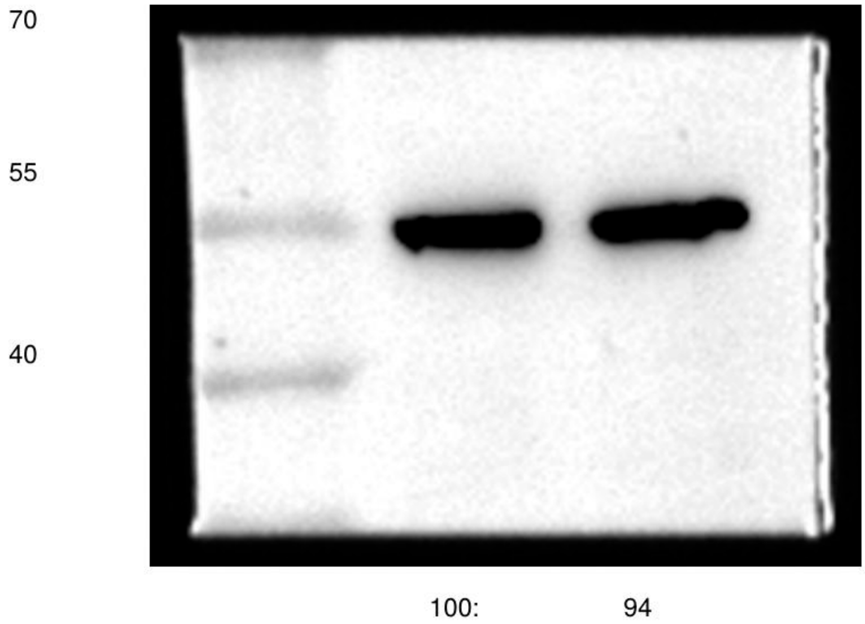

Original Image 32. Supplementary Figure S4B anti-EIF3H (shNC vs shEIF3H-1/2/3)

IB: anti-EIF3H (shNC vs shEIF3H-1/2/3)

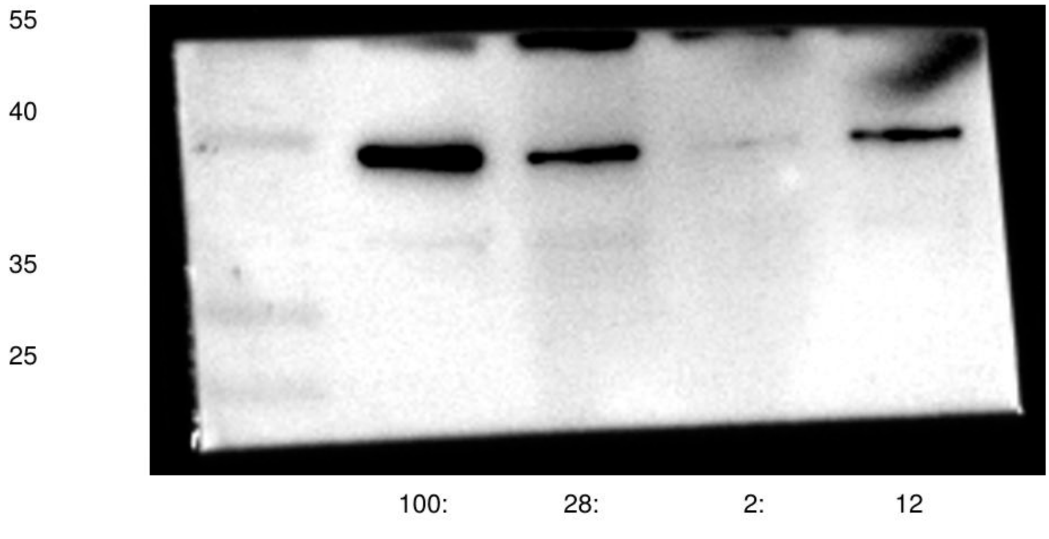

Original Image 33. Supplementary Figure S4B   anti- -Tubulin (shNC vs shEIF3H-1/2/3)

IB: anti-β-Tubulin (shNC vs shEIF3H-1/2/3)

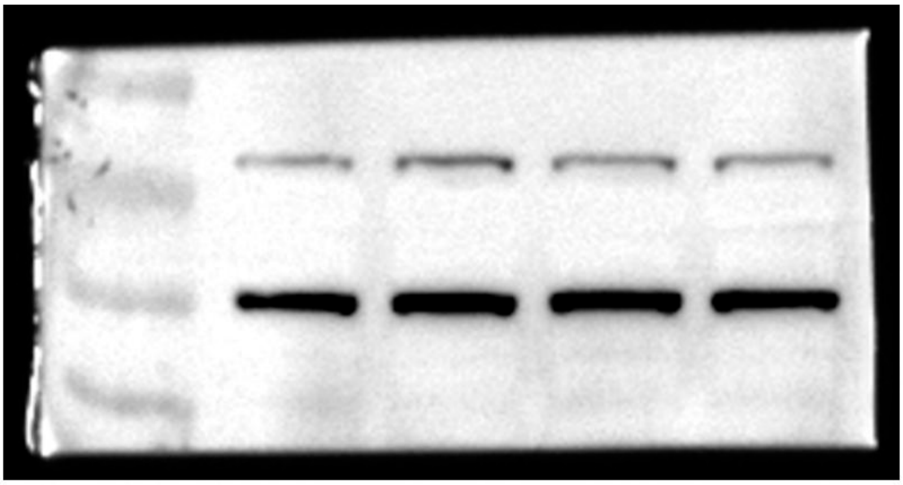

Original Image 34. Supplementary Figure S4C   anti-LDHA (shNC vs shLDHA-1/2/3)

IB: anti-LDHA (shNC vs shLDHA-1/2/3)

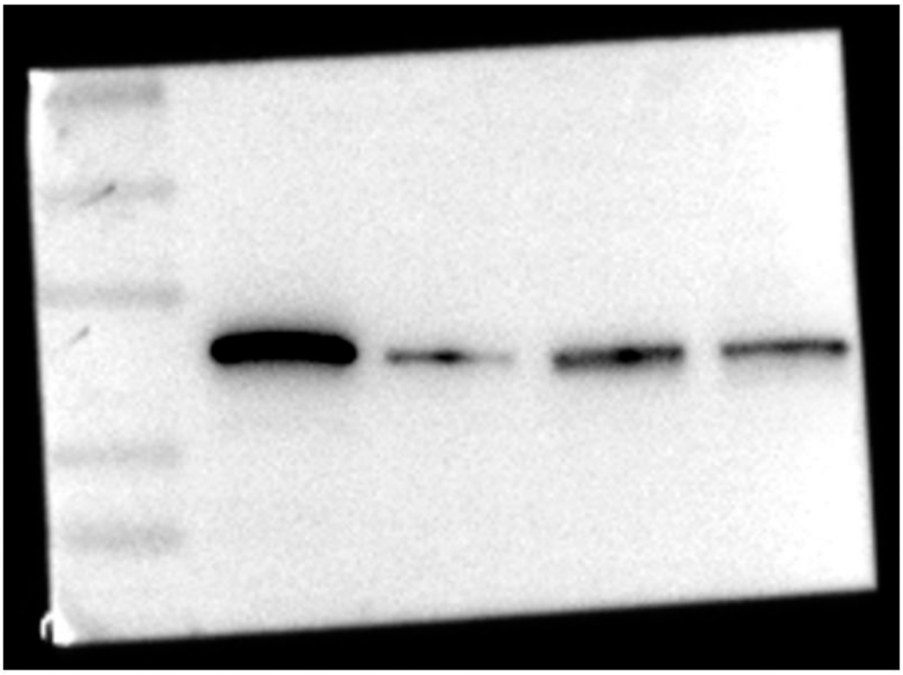

Original Image 35. Supplementary Figure S4C anti- -Tubulin (shNC vs shLDHA-1/2/3)

IB: anti-β-Tubulin (shNC vs shLDHA-1/2/3)

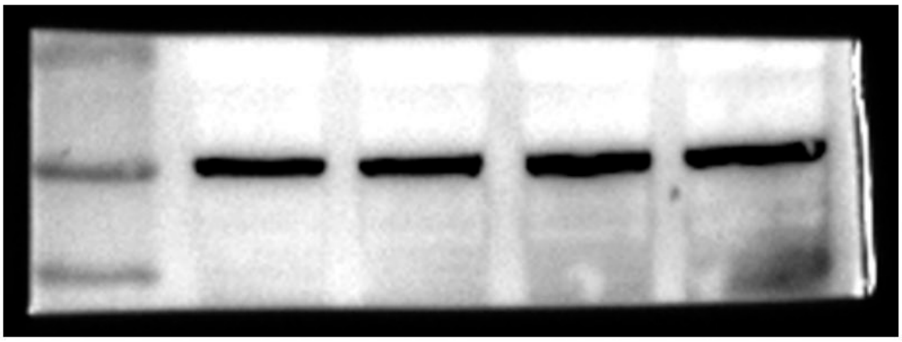

Original Image 36. Supplementary Figure S4D anti-GPR65 (shNC vs shGPR65-1/2/3)

IB: anti-GPR65 (shNC vs shGPR65-1/2/3)

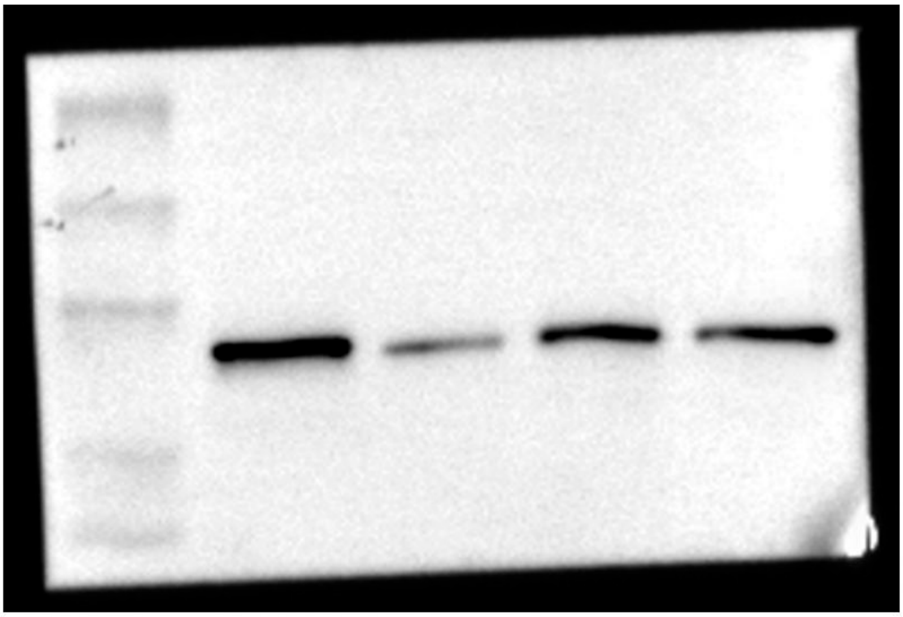

Original Image 37. Supplementary Figure S4D   anti- -Tubulin (shNC vs shGPR65-1/2/3)

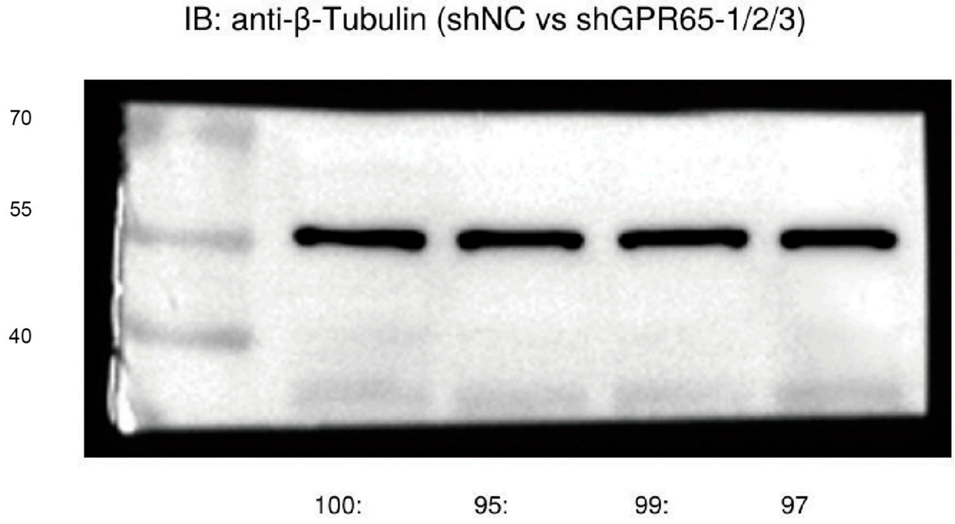

Original image 38. Supplementary Figure S1I anti-TRPS1(shNC vs shTRPS1-1/2/3 from lane5-8)

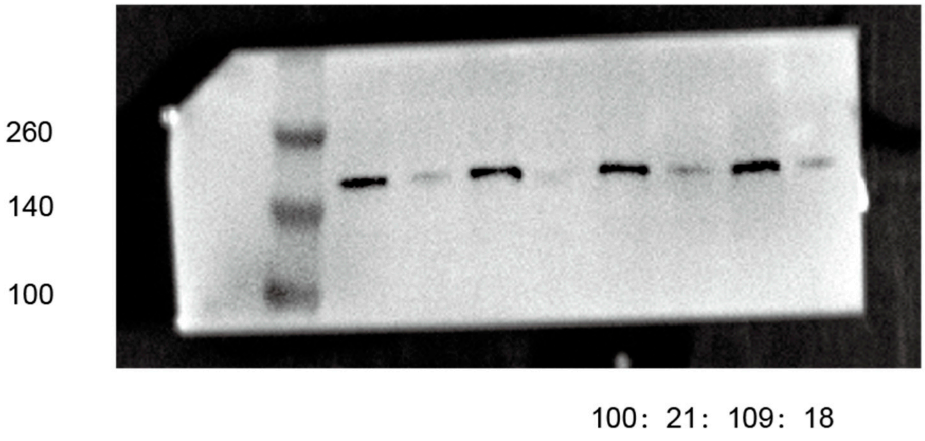

Original image 39. Supplementary Figure S1I anti-Tublin(shNC vs shTRPS1-1/2/3 from lane5-8)

70

55

40

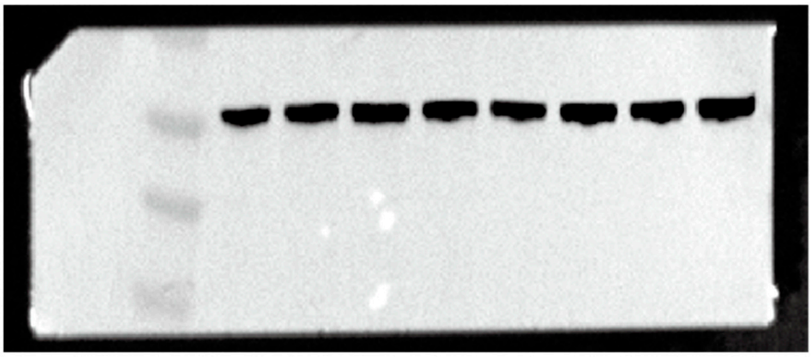

100：95：98：109

Original image 40. Supplementary Figure S4A anti-EIF3H(OE-NC vs OE-EIF3H)

55

40

35

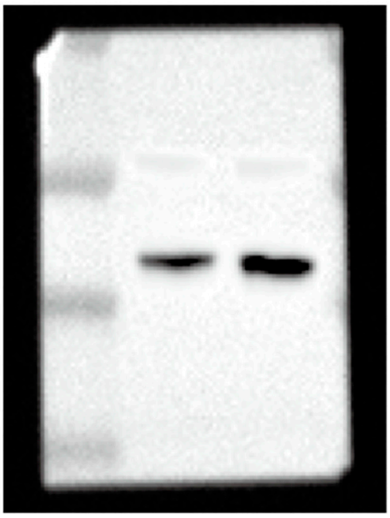

100：183

Original image 41. Supplementary Figure S4A anti-Tubulin(OE-NC vs OE-EIF3H)

70

55

40

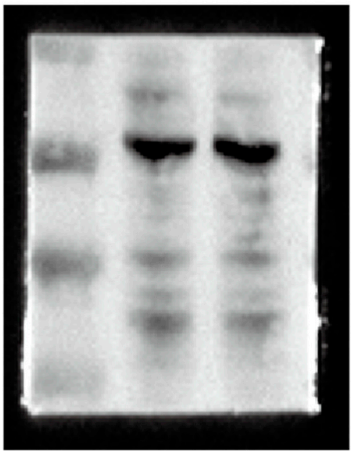

100: 109

Original image 42. Supplementary Figure S4B anti-EIF3H(shNC vs shEIF3H1/2/3)

55

40

35

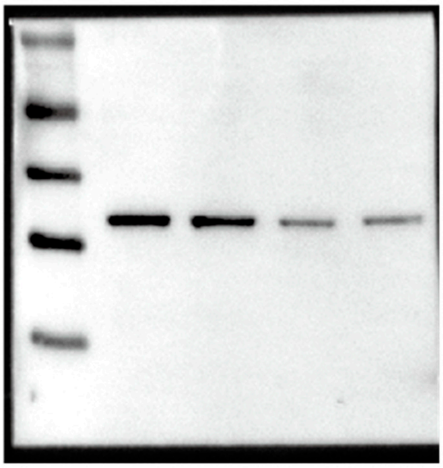

100: 86: 21: 23

Original image 43. Supplementary Figure S4B anti-Tubulin(shNC vs shEIF3H1/2/3)

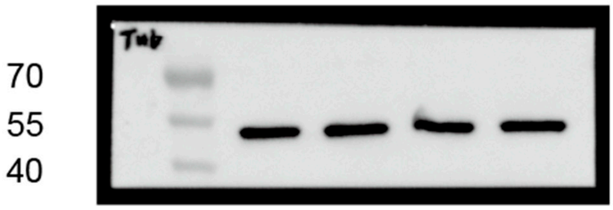

100: 105: 93: 102

Original image 44. Figure 1F EIF3H

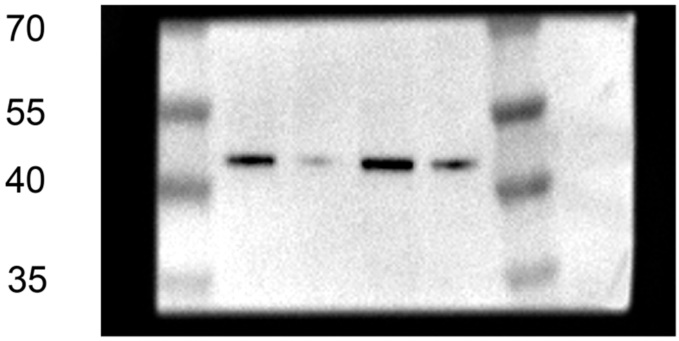

100: 15: 144: 83

Original image 45. Figure 1F tubulin

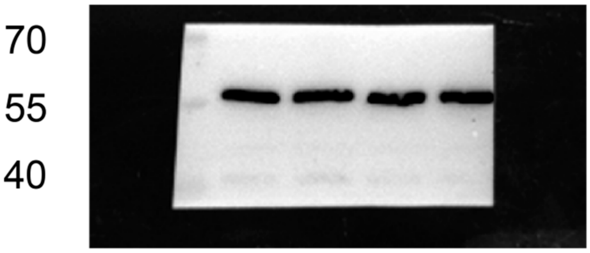

100: 96: 97: 104

Original image 46.Figure 1E EIF3H

70
55
40
35

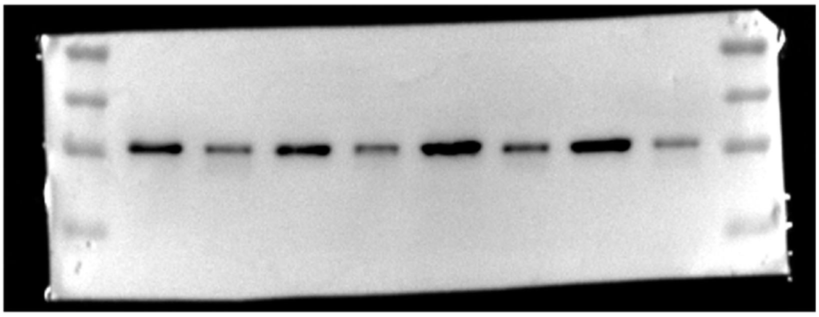

100：18：131： 13：152：24：138：16

Original image 47.Figure 1E Tubulin

70
55
40

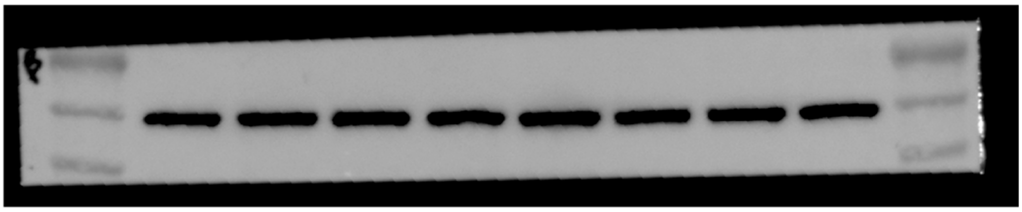

100：105：106：104：109：103：98：101

Table S1: Clinical characteristics of the patient cohort

Clinical features	
Age	
≤51	26（57.7）
>51	19（42.3）
T stage	
1	24（53.3）
2	16（35.4）
3	5（11.3）
N stage	
0	14（31.1）
1	15（33.3）
2	10（22.2）
3	6（13.3）
Menopausal status	
Yes	21（46.6）
No	24（53.3）

Table S2: List of genes correlated with EIF3H expression（UALCAN）

UALCAN-TNBC (EIF3H related)

Genes	PearsonCC
UTP23	0.57
EBAG9	0.44
RAD21	0.44
MRPL13	0.42
UBR5	0.40
TTC35	0.40
TRPS1	0.39
CPNE3	0.38
DCAF13	0.37
WDYHV1	0.37
POLR2K	0.37
FAM82B	0.36
NSMCE2	0.36
EIF3E	0.35
KIAA0196	0.35
MRPS28	0.35
TMEM70	0.35
COPS5	0.34
ZFAND1	0.34
ANKRD46	0.34
ZNF706	0.33
ATP6V1C1	0.33
TRMT12	0.33
TATDN1	0.33
MTDH	0.33
RNF139	0.32
JPH1	0.32
TERF1	0.32
C8orf76	0.32
RRM2B	0.32
DERL1	0.32
AZIN1	0.32
STAU2	0.32
NKIRAS1	0.32
IGFBP5	0.32
TPD52	0.32
UBE2W	0.31
SPAG1	0.31
ZNF252	0.31
ARFGEF1	0.31

RSL1D1	0.31
ESRP1	0.31
CRISPLD1	0.30
TCEB1	0.30
PEX2	0.30
TCEA1	0.30
IMPAD1	0.30
LACTB2	0.30
C8orf33	0.30
TAF2	0.30

Table S3: List of genes correlated with EIF3H expression (METABRIC)

METABRIC (EIF3H related)

Genes	Cytoband	SpearmanCC
RPL30	8q22.2	0.49
EBAG9	8q23.2	0.46
TATDN1	8q24.13	0.46
UTP23	8q24.11	0.46
WASHC5	8q24.13	0.45
ZFAND1	8q21.13	0.43
NTAQ1	8q24.13	0.43
PEX2	8q21.13	0.43
MAL2	8q24.12	0.41
MRPS28	8q21.13	0.41
CPNE3	8q21.3	0.41
LRATD2	8q24.21	0.40
ENY2	8q23.1	0.40
EEF1D	8q24.3	0.40
TMEM67	8q22.1	0.40
MRPL13	8q24.12	0.39
NBN	8q21.3	0.39
NSMCE2	8q24.13	0.39
RPS5	19q13.43	0.39
COPS5	8q13.1	0.39
NDUFAF6	8q22.1	0.38
RIDA	8q22.2	0.38
TAF2	8q24.12	0.38
RPSA	3p22.1	0.38
MED30	8q24.11	0.37
PCBD1	10q22.1	0.37
RPL27	17q21.31	0.36
TRPS1	8q23.3	0.36
NHP2	5q35.3	0.36
RSL1D1	16p13.13	0.36
PAIP2	5q31.2	0.35
RPLP0	12q24.23	0.35
OSGIN2	8q21.3	0.35
RPL12	9q33.3	0.35
ESRP1	8q22.1	0.35
TOMM20	1q42.3	0.35
MRPL45P2	17q21.32	0.35

CYC1	8q24.3	0.35
BTF3	5q13.2	0.34
E2F5	8q21.2	0.34
RPL24	3q12.3	0.34
FAM86B1	8p23.1	0.34
ANKRD46	8q22.3	0.34
GNL3	3p21.1	0.34
UBE2W	8q21.11	0.34
RPL18	19q13.33	0.34
IMPDH2	3p21.31	0.34
BCCIP	10q26.2	0.33
RPL8	8q24.3	0.33
MYC	8q24.21	0.33
MTERF3	8q22.1	0.33
RPL13AP6	10q25.2	0.33
RUVBL1	3q21.3	0.33
TRMT12	8q24.13	0.33
RPL17	18q21.1	0.33
NSA2	5q13.3	0.33
C1QBP	17p13.2	0.33
C11ORF1	11q23.1	0.33
TMEM69	1p34.1	0.32
PVT1	8q24.21	0.32
RPL13A	19q13.33	0.32
MCAT	22q13.2	0.32
CELSR1	22q13.31	0.32
MRPS23	17q22	0.32
CCT2	12q15	0.32
RPL12P38	17q23.2	0.32
RPL22	1p36.31	0.32
PHF20L1	8q24.22	0.32
PLEKHF2	8q22.1	0.32
RRS1	8q13.1	0.32
RPL7	8q21.11	0.32
NDUFAF2	5q12.1	0.32
NAA20	20p11.23	0.32
COX7C	5q14.3	0.31
DPH5	1p21.2	0.31
MRPL40	22q11.21	0.31
TIMM9	14q23.1	0.31
TCEA1	8q11.23	0.31
ZNF7	8q24.3	0.31
PPP1R12B	1q32.1	0.31
TBCA	5q14.1	0.31

SPCS1	3p21.1	0.31
RPS13	11p15.1	0.31
RMDN1	8q21.3	0.31
RRM2B	8q22.3	0.31
RPS16	19q13.2	0.31
RAN	12q24.33	0.31
NOA1	4q12	0.31
DCTPP1	16p11.2	0.31
RPS9	19q13.42	0.30
PTCD2	5q13.2	0.30
RPS20	8q12.1	0.30
TRIM49	11p11.12-q12	0.30
NUP35	2q32.1	0.30
PRMT5	14q11.2	0.30
PAX7	1p36.13	0.30
RPL35A	3q29	0.30
DERL1	8q24.13	0.30

Table S4: Intersection of EIF3H-correlated genes between UALCAN-TNBC and METABRIC cohorts

Intersection of UALCAN-TNBC and METABRIC (EIF3H related)

Genes	PearsonCC (UALCAN-TNBC)	SpearmanCC (METABRIC)	TF (Lambert et al.)
UTP23	0.57	0.46	No
EBAG9	0.44	0.46	No
MRPL13	0.42	0.39	No
TRPS1	0.39	0.36	Yes
CPNE3	0.38	0.41	No
NSMCE2	0.36	0.39	No
MRPS28	0.35	0.41	No
COPS5	0.34	0.39	No
ZFAND1	0.34	0.43	No
ANKRD46	0.34	0.34	No
TRMT12	0.33	0.33	No
TATDN1	0.33	0.46	No
RRM2B	0.32	0.31	No
DERL1	0.32	0.30	No
UBE2W	0.31	0.34	No
RSL1D1	0.31	0.36	No
ESRP1	0.31	0.35	No
PEX2	0.30	0.43	No
TCEA1	0.30	0.31	No
TAF2	0.30	0.38	No
